# Antimicrobial peptides: natural templates for next-generation therapeutics against antimicrobial resistance

**DOI:** 10.3389/fcimb.2025.1720027

**Published:** 2026-01-05

**Authors:** Ng Ngashangva, Surmani Huidrom, Indira Sarangthem Devi

**Affiliations:** Microbial Resources Division, Institute of Bioresources and Sustainable Development (IBSD) Takyelpat, Imphal, Manipur, India

**Keywords:** antibiotics, antimicrobial activity, antimicrobial peptides, antimicrobial resistance, antimicrobials

## Abstract

Antimicrobial resistance is a growing global health crisis, responsible for nearly five million deaths annually and projected to double by 2050 as conventional antibiotics fail against multidrug-resistant pathogens. AMR is aggravated by antibiotic misuse, weak regulations, inadequate prevention, high treatment costs, and the limited discovery of new antimicrobials. In this context, antimicrobial peptides, including natural, synthetic, and computationally designed variants, have emerged as promising alternatives. AMPs display broad-spectrum antibacterial, antifungal, antiviral, antiparasitic, antibiofilm, and immunomodulatory activities, with a lower tendency to induce resistance. Their mechanisms include membrane disruption, intracellular targeting, immune modulation, and selective binding to negatively charged microbial membranes. Structural features such as α-helices, β-sheets, cyclic motifs, and post-translational modifications enhance potency and specificity. Recent advances in chemical modification, recombinant expression systems, nanotechnology, and AI-driven computational approaches have improved AMP stability, bioavailability, and therapeutic efficacy. Synthetic derivatives like innate defense regulators and conjugated AMPs further enhance immunomodulatory properties and reduce toxicity, while combination therapies increase effectiveness. Challenges remain, including degradation, short half-life, production costs, and microbial defenses such as biofilms and efflux pumps. Nevertheless, high-throughput sequencing and screening, structural biology, and structure–activity relationship studies continue to accelerate AMP development, positioning them as vital next-generation therapeutics against AMR.

## Introduction

The discovery of antibiotics revolutionized medicine, extending human life expectancy and transforming the treatment of infectious diseases (Harvey et al., 2015). However, as Fleming warned, the efficacy of penicillin would be temporary, and today antimicrobial resistance (AMR) has emerged as a critical clinical and public health crisis (Fleming, 1929; ECDC, 2013). The World Health Organization (WHO) describes AMR as a “silent pandemic” and “ticking time bomb,” attributing its rise to antibiotic misuse, weak regulatory frameworks, inadequate preventive measures, high medication costs, lack of new antimicrobial, and inequitable access to clean water (WHO, 2014). In 2019, resistant bacterial infections were associated with nearly 4.95 million deaths; including 1.27 million directly attributed to AMR, and imposed an economic burden exceeding $1.7 billion USD (WHO, 2022). By 2050, annual deaths could reach 10 million, with a projected global economic loss of $134 trillion, reducing gross domestic product (GDP) by 2-3.8% (Antimicrob Res Collab, 2022). Conversely, delaying resistance by even a decade could generate $65 trillion in savings between 2014 and 2050 (Antimicrob Res Collab, 2022).

The burden of AMR is unevenly distributed, with low- and middle-income countries (LMICs) disproportionately affected due to unregulated antibiotic sales, inappropriate prescribing, and extensive use of antimicrobials in agriculture and industry. These factors, along with globalization, traveling, food chain transmission, and climate change, accelerate the spread of resistant microbes (Morar and Wright, 2010; Wellington et al., 2013; WHO, 2022). Vulnerable populations such as children under five, the elderly, neonates, and immunocompromised individuals face the highest risks (Morgan et al., 2011). Seven bacterial groups-*Enterococcus faecium*, *Staphylococcus aureus*, *Klebsiella pneumoniae*, *Acinetobacter baumannii*, *Pseudomonas aeruginosa*, and *Enterobacter* species (ESKAPEE pathogens) account for over 70% of AMR-associated deaths, overlapping with the WHO priority pathogens list (Antimicrob Res Collab, 2022).

Resistance against antibiotics arises through intrinsic microbial traits or adaptive, acquired, and phenotypic mechanisms (Hughes and Andersson, 2015; Blair et al., 2014). Environmental dissemination of antibiotics *via* agriculture, industrial waste, and animal husbandry amplifies selection pressure and accelerates resistance (DCosta et al., 2011; Berendonk et al., 2015). As a result, AMR undermines medical interventions, including surgery, organ transplantation, cancer therapy, and HIV treatment (WHO, 2022). Meanwhile, the discovery of new antibiotics has declined, hindered by long development timelines, rapid resistance emergence, and poor commercial incentives (Schaberla and Hack, 2014).

In this context, antimicrobial peptides (AMPs) have gained increasing attention (Kaspar and Reichert, 2013). In 2020, the global AMP market was valued at $5 million and is projected to reach $6 million by 2027, representing a compound annual growth rate (CAGR) of 5.4% (Uhliga et al., 2014; Global Antimicrobial Peptides Sales Market Report, 2021; Transparency Market Research, 2012). AMP-based therapeutics are promising against AMR due to their slower resistance development, broad-spectrum activity, potent antimicrobial effects, including efficacy against multidrug-resistant (MDR) and ESKAPEE pathogens and antibiofilm properties, targeting biofilm which is responsible for 65% of infections (Fosgerau and Hoffmann, 2015; Brown and Wright, 2016; Shriwastav et al., 2025; Malmsten, 2014; de la Fuente-Nunez et al., 2023). They act on multiple cellular components, such as membrane integrity, cell wall biosynthesis, and cytoplasmic processes, individually or in combination, while minimizing damage to non-target cells (Magana et al., 2020). Structurally, AMPs possess large surface areas, high chirality, and complex conformations amenable to genetic engineering and chemical synthesis, and their resemblance to endogenous metabolites facilitates active transport and enhances biological effects, including immune modulation (Ganesan, 2008).

Despite these advantages, AMPs face challenges such as low oral bioavailability, poor plasma stability, short circulation times, hemolytic effects, and delivery limitations (Muttenthaler et al., 2021). Orally administered peptides typically have half-lives of less than 30 minutes due to degradation by gastrointestinal enzymes and efflux pumps (Asif et al., 2024), and most AMPs current applications are topical (Cesaro et al., 2023). Strategies like amino acid modifications and chemical synthesis are being explored to enhance stability, prolong half-life, improve delivery, and minimize toxicity (Price and Patel, 2023).

A comprehensive understanding of AMPs’ mechanisms of action, structural and physicochemical properties, biosynthetic pathways, metabolism, delivery methods, and resistance mechanisms is crucial for optimizing therapeutic potential. AMP databases provide curated information on applications, efficacy against pathogens, and mechanistic insights to guide rational design against AMR (Lee et al., 2022; Epand and Vogel, 1999). The vast diversity of known and unexplored organisms, including extremophiles and species whose genomes may not have been exposed to antibiotics underscores the importance of natural AMP templates, yet only an estimated 3% of bacterial natural product biosynthetic pathways have been experimentally characterized, highlighting untapped potential for novel therapeutics (Newman and Cragg, 2020; Davies, 2011). Structure–activity relationships (SARs) studies of AMPs hold great potential, as their biological properties are basically determined by their structure, making them valuable natural templates to design AMR drugs.

Advances in computational deep learning, artificial intelligence (AI), and high-throughput experimental platforms have significantly accelerated the discovery and chemical modification of AMPs for preclinical and clinical applications (Magana et al., 2020; Stokes et al., 2020; Cresti et al., 2024; Cesaro et al., 2025; Zhang, 2025). Complementing these approaches, breakthroughs in whole genome sequencing (WGS), proteomics, and metabolomics have enabled identification of novel peptides and metabolites, while supporting advanced diagnostic development (van der Hooft et al., 2020). These technologies have strengthened surveillance and facilitated detection and characterization of antimicrobial resistance genes (ARGs) (Hendriksen et al., 2019). WGS provides single-nucleotide resolution for AMR gene detection, pathogen typing, virulence factor characterization, and tracing genetic ancestry (Pillay et al., 2022). WGS-based antimicrobial susceptibility testing (AST), exemplified by the ResFinder tool, generates reliable *in silico* antibiograms comparable to conventional phenotypic methods (Zankari et al., 2012).

Beyond laboratory research, effective strategies to combat AMR must encompass strengthening health surveillance, promoting responsible antibiotic use, enhancing resistance monitoring, and advancing antimicrobial stewardship and infection control at local and national levels. In countries like India, educational programs targeting schools, communities, healthcare workers, professionals, drug manufacturers, vendors, and the public can play a critical role in these interventions. The growing threat of AMR endangers the achievement of global Sustainable Development Goals (SDGs), underscoring the need for interdisciplinary research and coordinated actions to guide global priority setting, inform public health policies, and support evidence-based treatment decisions and reinforced with WHO One Health initiative (WHO, 2022).

In this study, we highlight the health and economic burden of AMR, AMP sources, structural properties, mechanisms, limitations, examine their current clinical applications and rediscovering their potentials. We examine the SARs studies that identify structural features that govern its biological activities. We further explore how integrating omics datasets with cutting-edge AI and machine learning (ML) can accelerate AMP discovery and development, positioning peptides as effective alternatives to counter resistant.

This review distinguishes itself from earlier work by offering a comprehensive, integrative examination of AMPs as sustainable, natural product-based solutions to combat AMR. While previous reviews often address isolated aspects of AMP biology, this study considers AMPs holistically as natural molecules, as modifiable templates for high-throughput innovation, and as entities increasingly advanced by modern omics- and AI-driven discovery tools. Developed over the past decade (2015–2025), it aligns with the One Health framework and synthesizes the key attributes of AMPs needed to guide the rational design of next-generation therapeutics against AMR.

## Literature search methodology

A structured literature search was conducted using PubMed, Google Scholar, and Scopus to identify peer-reviewed publications relevant to antimicrobial resistance (AMR), antimicrobial peptides (AMPs), structure–activity relationship (SAR) studies, delivery technology, and advances in omics and artificial intelligence–driven peptide discovery. Searches were performed using combinations of keywords such as “antimicrobial peptides against antimicrobial resistance”, “antimicrobial peptides,” “AMR,” “structure–activity relationship,” “peptide stability,” “WGS-based AMR detection,” “AI and ML in AMPs drug discovery,” and “peptide in therapeutic applications.” The initial search retrieved 1,298 publications. After an assessment of titles and abstracts, 700 papers were excluded for not meeting the study scope, and 42 papers related to AMPs applications which are not align with our manuscript were excluded. An additional 268 articles were removed during full-text screening based on predefined exclusion criteria, resulting in 288 studies being included in the final synthesis.

### Exclusion criteria comprised

- Non-original research (e.g., commentaries, editorials, conference abstracts).
- Irrelevant focus, including studies unrelated to AMPs, AMR, SAR, peptide design, or omics technologies.
- Insufficient methodological detail or absence of mechanistic or experimental data.
- Duplicate publications across search engines.
- Non-English language articles, due to limits in standardized interpretation.

This approach ensured that only high-quality, relevant, and methodologically robust studies were included to support the analysis of AMPs, their mechanisms, limitations, and the emerging role of computational and omics technologies in peptide-based drug development.

## Source of peptides

The study of biologically active peptides dates back to the early 20^th^ century. In the late 1920s, Alexander Fleming discovered lysozyme, the first peptide reported with antimicrobial activity (Fleming, 1929), followed by René Dubos’ isolation of gramicidin from a soil *Bacillus* strain in 1939, marking the first clinically tested antibacterial peptide effective against pneumococcal infections in mice (Lopes et al., 2022). In the 1980s, Hans Boman identified an induced cecropin from the hemolymph of *Hyalophora cecropia* pupae (Steiner et al., 1981), and subsequently, AMPs were gradually discovered across multiple phyla and environments (Haas et al., 1938; Dimarcq et al., 1998; Tomasz, 2006; Maroti et al., 2011; Jansson and Tas, 2014; Mathieu et al., 2017) (**Supplementary Table S1**). As of January 2025, the APD3 database (https://aps.unmc.edu/) catalogs 5,099 peptides, including 3,306 natural AMPs from six kingdoms-410 bacterial bacteriocins, 5 from archaea, 8 from protists, 29 from fungi, 268 from plants, and 2,580 from animals- along with 1,299 synthetic and 231 computational predicted AMPs (Wang et al., 2016) (**Table 1**) (**Supplementary Figure S1**). Although peptides are highly conserved, their evolutionary lineage across kingdoms remain incompletely understood (Vizioli and Salzet, 2002; Huan et al., 2020). These naturally encrypted peptides are valuable natural templates for alternative antibiotics and bioinspired engineered therapies (Torres et al., 2021).

**Table 1:** Peptide based drugs from diverse sources, along with their structures, modes of action, and activities against AMR pathogens.

Peptide	Structure	Source	Mode of action	Activity
Defensing	β-sheet	Human neutrophil, rabbit, primate	Membrane permeabilization and/or lysis	*Candida ablicans*, anti-enveloped viruses, Anticancer, Broad-spectrum antibacterial, wound healing, Antiparasite, Immune defense, *Staphylococcus aureus*
Magainin	α-helix	*Xenopus laevis*	Permeabilizes bacterial membrane	*C. ablicans*, spermicidal
Indolicidin	Non-αβ	Bovine neutrophil	Membrane permeabilization activity	*Escherichia coli, Pseudomonas aeruginosa, Salmonella typhimurium*
Cecropin	α-helix	*Chlamydia trochomatis*	Membrane destabilizing	Cytotoxic to pathogens, HIV
Cathelicidins	Amphipathic α-helices	*Treponema palladium*, animals neutrophils	Disintegration of cell membrane	Inhibits multiplication of pathogens
Nisin	Cationic	Bacteriacea	Inhibition of cell wall synthesis	*P. aeruginosa*, increase cellular immunity, *Helicobacter pylori*, and oral mucositis
Bacteriocins	Amphiphilic α- helix	Bacteria	Pore forming	Related bacterial strains, *E. coli*
Alamethecin	α-helix	*Trichoderma viride*	Channel forming	Antimicrobial
Gramicidin S	Antiparallel β-sheet	*Bacillus brevis*	Cell Permeation	Spermicide, Sexually transmitted disease
Plant defensing (thionins)	hairpin-like helical peptide	Plants	Cell Permeation	Antifungal, Inhibits phytopathogenic bacteria
Mellitin	α-helix	Insects	Membrane destabilizing, Cellular target	Anticancer, Antivenom
Drosomycin	α-helical and β-sheet	Fly	Hyphae lysis	Antifungal, Toll signal pathway, Innate immunity

AMPs are produced through diverse biosynthetic pathways involving post-translational modifications (PTMs) (Wang, 2012). Non-ribosomal peptides (NRPs) exhibit heightened resistance to hydrolases and enhanced *in vivo* stability compared to ribosomally synthesized peptides (Finking and Marahiel, 2004; Arnison et al., 2013). In multicellular organisms, ribosomally synthesized AMPs such as cathelicins are induced as part of the innate immune response *via* gene transcription and translation (Ganz, 2003). NRPs, including bacteriocins, are typically synthesized as prepropeptides with an N-terminal signal sequence, pro-segment, and C-terminal cationic peptide, often containing lanthionine, h-methyllanthionine, and other modifications, forming lantibiotics which further classified into five classes (Katz and Demain, 1997). Proteolytic cleavage of larger proteins also yields cryptic AMPs with distinct functions, contributing to their structural diversity, often augmented by non-proteinogenic amino acids and extensive PTMs (Arnison et al., 2013; Wang, 2012). Most extensively studied NRPs originate from bacteria and fungi, which produce secondary metabolites (SMs) in ecological niches to compete for resources (Seiple et al., 2016; Rosenblueth and Martinez-Romero, 2006; Friesen et al., 2011; Klaenhammer, 1988; Riley and Wertz, 2002). Paired omics datasets, peptidogenomics, metabolomics, and high-throughput ML tools are increasingly used to integrate genomic and metabolomic data, enabling novel peptide discovery, improved metabolite annotation, expanded peptide libraries, efficient dereplication, and streamlined prioritization workflows for antimicrobial development (Schorn et al., 2021; Srinivas et al., 2010). Deep learning models now further enable *de novo* design of peptides, accelerating AMP discovery (Siegers et al., 1996).

Plant-derived AMPs were first exemplified by purothionin from wheat flour (Jenssen et al., 2006), and subsequently, several families have been identified, including thionins, defensins, lipid transfer proteins, hevein-like peptides, knottin-type peptides, α-hairpins, snakins, and cyclotides (Höng et al., 2021; Shanmugaraj et al., 2021). These peptides exhibit antibacterial, antifungal, insecticidal, and anticancer activities and are widely used in commercial biopesticides (Fritig et al., 1998; Nawrot et al., 2014). Amphibians, particularly frogs of genera *Xenopus*, *Silurana*, *Hymenochirus*, and *Pseudhymenochirus* (Pipidae family), produce AMPs that protect against pathogens (Barra and Simmaco, 1995; Huan et al., 2020). Insects also provide AMPs such as magainins and defensins, synthesized primarily in fat bodies and blood cells (Bulet et al., 1999; Torrent et al., 2012). Microbial sources include unculturable soil bacteria, exemplified by teixobactin, which shows potent activity against *Mycobacterium tuberculosis* and methicillin-resistant *Staphylococcus aureus* (MRSA) with low cytotoxicity and minimal resistance development (Ling et al., 2015). Additional sources include synthetic peptide libraries and *in silico* mutagenesis (Zhang et al., 2021), while ML approaches can identify potential AMPs from de-extinct genomes and optimize peptide activity and selectivity (Szymczak et al., 2023).

## Properties of AMPs

The AMP family encompasses a diverse range of functions, commonly referred to as cationic host defense peptides (CHDPs), and derived from various sequences and organisms. They primarily target microbial cell membranes, where mutations and resistance development are less frequent compared to conventional antibiotics (Peschel and Sahl, 2006). Typically, AMPs are small peptides, averaging fewer than 100 amino acid residues, and carry a net charge of +2 to +9 (Huan et al., 2020). Their structural diversity, shaped by PTMs, enhances the spectrum of antimicrobial activity (McIntosh et al., 2009). Within the APD3 database, AMPs are categorized based on biosynthetic mechanism, origin, function, covalent bonding pattern, three-dimensional (3-D) structure, biological activity, and molecular target (Wang, 2015). They range in length from 2 to 183 residues (average 33.27), with the majority (88.5%) between 11 and 60 residues. Most AMPs (96.2%) have a net charge between -5 and +10 (average +3.33) and a high proportion of hydrophobic residues (typically 50%) (Wang et al., 2016). Interestingly, some neutral and anionic AMPs exhibit distinct behaviors, avoiding membrane modification and resistance development (Dennison et al., 2018).

AMPs are cationic, amphiphilic, and membrane-permeabilizing, with amphipathicity being essential for interaction with pathogen membranes (Reddy et al., 2004; Shai, 2002; Zhang et al., 2021). This structural balance enables solubility in aqueous environments and interaction with negatively charged microbial surfaces (Zasloff, 2002; Gan et al., 2021). Hydrophobicity facilitates membrane permeabilization, though excessive levels can induce mammalian cell toxicity and reduce selectivity (Chen et al., 2005). Most AMPs (98.6%) have hydrophobicity between 10 and 80%, peaking at 40-50%. The antimicrobial activity of AMPs is pH-dependent and varies with sequence, mechanism, and pathogen cell wall composition. For Gram-negative bacteria (GNB), activity typically decreases with increasing pH (pH 4-9), whereas for Gram-positive bacteria (GPB), higher pH can enhance potency (Walkenhorst et al., 2013; Wimley, 2010).

The cationic nature of AMPs promotes accumulation near negatively charged bacterial surfaces, which differ between Gram-negative and Gram-positive species (Malanovic and Lohner, 2016). AMPs target negatively charged cell wall components such as teichoic and lipoteichoic acids in GPB, deterring resistance development (Papagianni, 2003; Huang, 2006; Silva et al., 2016). Bacterial membranes rich in phosphatidylglycerol (PG) attract cationic AMPs, whereas eukaryotic membranes composed mostly of neutral lipids like phosphatidylcholine (PC) and sphingomyelin (SM), limit peptide binding (Yang et al., 2001). Similarly, cancer cells often exhibit negatively charged outer membranes due to loss of membrane asymmetry, explaining AMP activity against tumor cells (Mader and Hoskin, 2006).

AMPs exhibit broad-spectrum antimicrobial activity, contrasting with the specificity often sought in novel therapeutics. Their versatility, ability to target pathogen cell wall components, and low tendency to induce resistance make them promising candidates against ESKAPEE pathogens. They also demonstrate efficacy against GPB, GNB, fungi, viruses, and cancer cells, particularly in contexts where drug resistance is pronounced.

## Structure of AMPs

**Figure 1:**
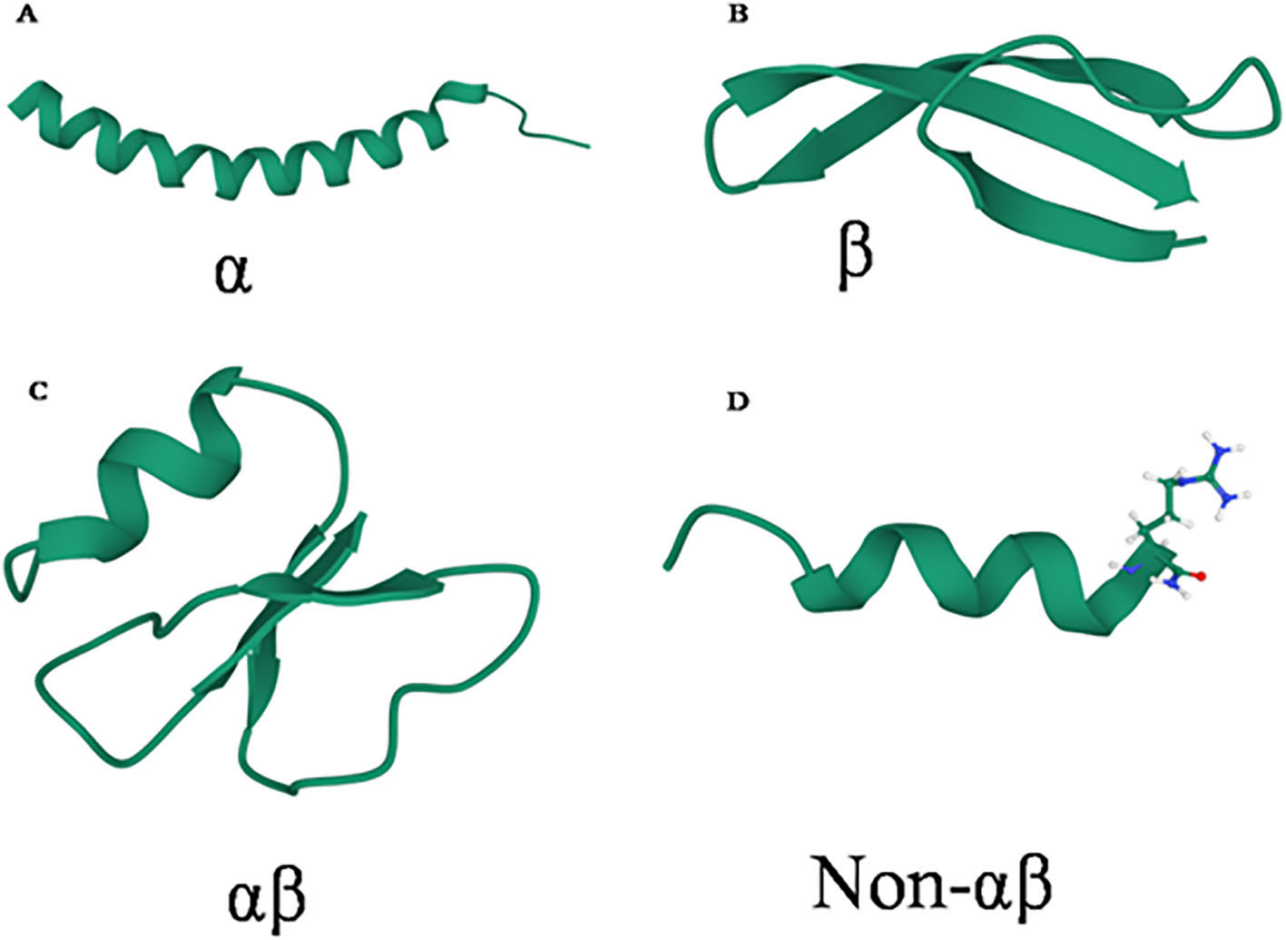
Schematic representation of 3-D structure of antimicrobial peptides shown are representatives from each family. **(A)** α-helical structure of human cathelicidin LL-37 (PDB entry: 2K6O) (Wang, 2008) **(B)** the β-sheet structure of Human defensin 5 (PDB entry: 2LXZ) (Wommack et al., 2012) **(C)** the αβ structure of avian β-defensin (PDB entry: 6QES) (Guyot et al., 2020); and **(D)** the non-αβ structure of cattle indolicidin peptide derivative with P–>A substitution (PDB entry: 1HR1) (Friedrich et al., 2001; Wang et al., 2016; Sehnal et al., 2021).

Ribosomally synthesized natural AMPs are classified into major families based on their secondary structures: alpha-helical (α), beta-sheet (β), alpha-beta (αβ), mixed α-helical and β-sheet, non-αβ, and cyclic conformations, with classification influenced by the number, spacing, and connectivity of cysteine (Cys) residues (Wang, 2008) (**Figure 1**). Representative examples include the α-family peptide LL-37 (cathelicidin), the β-family peptide human defensin 5, the αβ-family avian β-defensin, and non-αβ peptides such as cattle indolicidin (Wang, 2008; Frasca and Lande, 2012; Wang et al., 2016). AMPs are structurally flexible and can adopt different conformations upon interacting with microbial membranes, transitioning from unfolded forms to α-helices or β-sheets, which facilitate alignment with lipid regions, pore formation, and microbial cell disruption- a feature that can be exploited in structure-based drug design (Wang, 2015; Oliveira Júnior et al., 2025).

The structural and functional properties of AMPs have been extensively characterized using solid-state Nuclear Magnetic Resonance (NMR) spectroscopy, X-ray crystallography, and computational approaches including molecular modeling, docking, and molecular dynamics simulations (Marion et al., 1988; Schibli et al., 2006; Holak et al., 1988). Conserved structural motifs, such as α-helices, β-sheets, β-turns, and γ-turns, are critical for receptor recognition, antimicrobial potency, selectivity, and proteolytic stability (Muttenthaler et al., 2021). For example, the α-helical AMP buforin II, derived from frog histones, interacts with microbial membranes *via* electrostatic and hydrophobic interactions, and then accumulates intracellularly to inhibit essential processes in *E. coli* (Wimley, 2010). In contrast, β-sheet AMPs form oligomeric transmembrane β-barrels in anionic membranes or β-sheet aggregates on cholesterol-containing membranes (Wang et al, 2016). Certain extended AMPs, such as drosocins, lack regular secondary structures but are enriched in arginine (Arg), tyrosine (Tyr), and proline (Pro) residues, illustrating functional diversity (Nguyen et al., 2011).

However, not only the secondary structure of AMPs, the antimicrobial activity of AMPs is influenced by multiple factors, including a threshold cationic charge, sufficient local concentration, the capacity for self-assembly or multimerization, and the composition, fluidity, and phospholipid head group size of microbial membranes (Yeaman and Yount, 2003). Leveraging the structural versatility of AMPs remains an underexplored strategy that could advance the rational design of peptide-based therapeutics and enhance their efficacy against AMR pathogens.

## Mechanism of action of AMPs

Biochemical studies have revealed AMP interactions with microbial targets, including ligands and receptors, and demonstrated that cationic AMPs, enriched in Arg and Lys residues, preferentially bind to negatively charged bacterial membranes through electrostatic interactions. Structural features such as the N-terminal free amino group and C-terminal amidation enhance binding, net charge, stability, and membrane activity (Hancock and Diamond, 2000; Hasper et al., 2006; Epand and Vogel, 1999). Fungal and parasitic AMPs, like ceropin and cathelicidin, penetrate membranes to interact with intracellular proteins, including heat-shock proteins (hsp), and inhibit ATPase activity (Pitale et al., 2020). Depending on their targets, AMPs are classified as antibacterial, antifungal, antiviral, or antiparasitic, though many display broad-spectrum activity, highlighting their potential against MDR pathogens.

**Figure 2:**
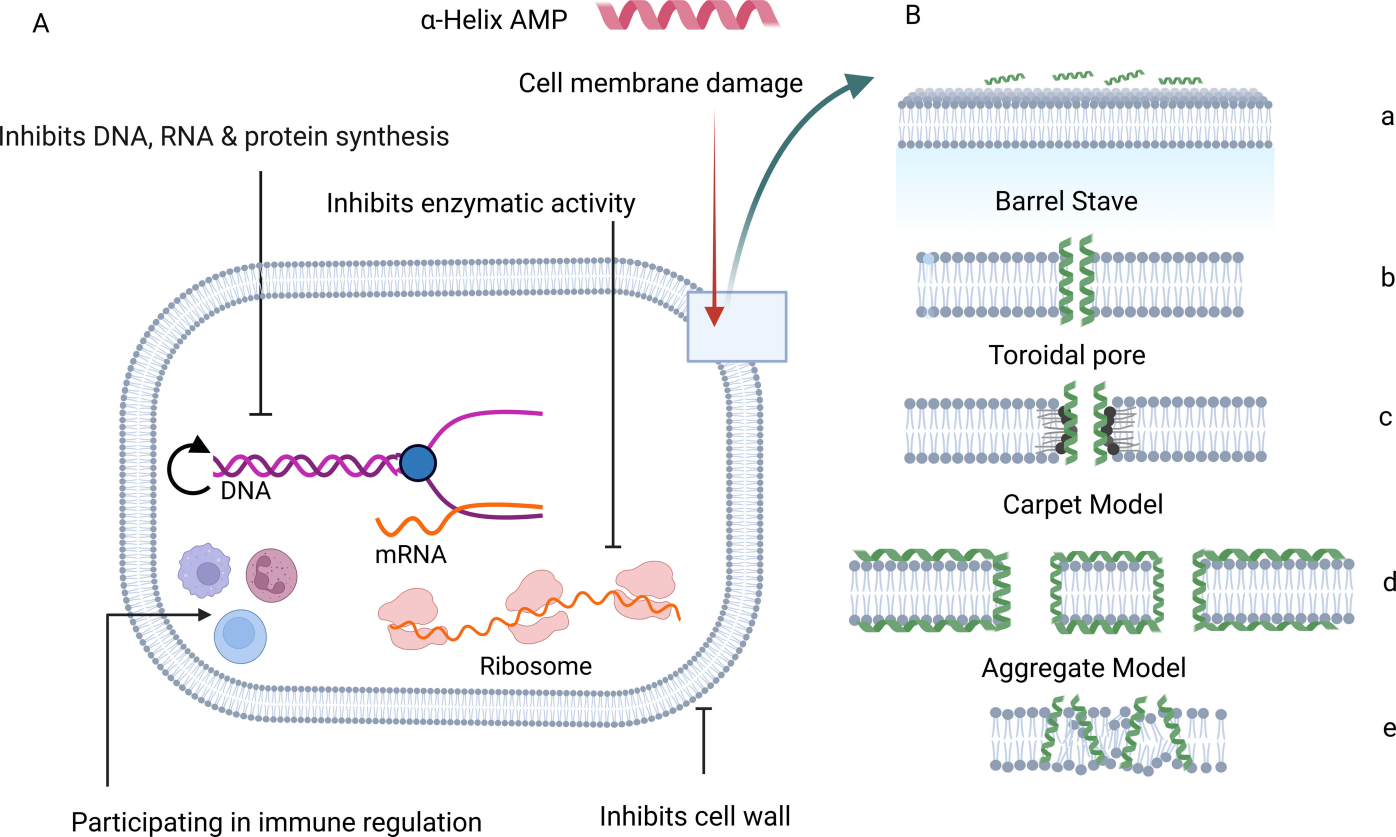
Schematic diagram of antimicrobial peptide mode of action models with mechanisms for interaction of AMPs with bacterial cell membranes using X-ray electron density and neutron studies. **(A)** The mechanism of action of AMP is through cell membrane damage, intracellular bactericidal mechanism, inhibition of the synthesis of macromolecules, damage of the organelles to cause DNA fragmentation, inhibition of enzyme activity, and antimicrobial effect via participating in immune regulation. **(B)** (a) Cell membrane and AMP (b) Barrel-stave model, (c) Toroidal pore model, (d) Carpet model, and (e) Aggregate model (Huang, 2006). (Image: Created in BioRender.com).

In GNB, cationic AMPs such as MSI-78, a synthetic magainin 2 analogue, initially interact with lipopolysaccharides (LPS) to facilitate penetration into the inner membrane, disrupting intracellular components and inducing leakage that leads to cell death (Hallock et al., 2003; Bechinger and Gorr, 2017). Beyond membrane disruption, AMPs act intracellularly to inhibit DNA replication, RNA transcription, ribosome translation, or other essential processes (Brogden, 2005; Zhang et al., 2021). Their antimicrobial effectiveness depends on factors such as peptide concentration, conformational changes, peptide-to-lipid ratio, and the composition, fluidity, and headgroup size of microbial membranes (Hasper et al., 2006; Yeaman and Yount, 2003) (**Figure 2**). Some AMPs also enhance host defenses by modulating immune components, indirectly contributing to pathogen clearance. Importantly, the nonspecific electrostatic and hydrophobic interactions with bacterial membranes reduce the likelihood of resistance development, distinguishing AMPs from conventional antibiotics, which target specific metabolic pathways (Epand and Vogel, 1999; Witek et al., 2017).

Four principal models describe AMP-induced membrane perturbation: barrel-stave, toroidal pore, carpet, and aggregate models (Shai, 2002). In the barrel-stave model, peptides insert and aggregate within oriented lipid bilayers, forming pores as observed for alamethicin (Huang, 2006; Mohr and Kleinkauf, 1978). Toroidal pore formation, exemplified by magainins, aurein 2.2, melittin, nisin, and colistin, occurs when peptides induce lipid monolayers to bend through the pore, with both peptides and lipid headgroups lining the water core (Matsuzaki et al., 1989; Hancock and Lehrer, 1998; Mihajlovic and Lazaridis, 2010). The carpet model involves parallel peptides adsorption onto the membrane, generating detergent-like effects that form micelles and disintegrate membranes, demonstrated in dermaseptin and insect-derived peptides (Huang, 2006; Rozek et al., 2000). In the aggregate model, peptides cluster on or within membranes, forming transient pores that promote cytoplasmic leakage (Teixeira et al., 2012). Although these models are grounded in the fluid mosaic framework, they do not fully capture all AMP actions, highlighting the need for further mechanistic studies.

Experimental techniques for elucidating AMP mechanisms include live-cell imaging, artificial membrane assays, microscopy with labeled peptides, circular dichroism (CD) spectroscopy for peptide folding, dual-polarization interferometry for lipid ordering, X-ray crystallography, neutron diffraction, and vesicle permeabilization assays (Kelly and Price, 2000; Matsuzaki et al., 1989; Wommack et al., 2012; Wang, 2015; Fani et al., 2012). For example, specific cationic pro-rich peptides penetrate bacterial membranes and disrupt intracellular targets, while others like edeine, tuberactinomycins, and dityromycin inhibit ribosomal protein biosynthesis, reflecting the possibility of multiple, overlapping mechanisms for a single AMP (Brogden, 2005; Bechinger, 1999). Despite their diverse modes of action, AMPs primarily target microbial membranes without cross-resistance, making them promising candidates for novel anti-AMR therapeutics.

## Antiviral activity

The escalating resistance among viruses, combined with the pandemic risk posed by emerging pathogens such as coronavirus 2 (SARS-CoV-2), Dengue, Chikungunya, Ebola, Mpox, and Zika, underscore the urgent need for effective antiviral therapeutics (Chowdhury et al., 2020; Ashaolu et al., 2021; Mora et al., 2022). Conventional antiviral drugs often face limitations including emerging resistance, suboptimal efficacy, and side effects. In contrast, antiviral peptides (AVPs), a specialized subset of AMPs, provide a promising alternative due to their potential for chemical and biological modifications, enabling enhanced therapeutic design and reduced viral resistance (Vilas et al., 2019). AVPs act by disrupting viral replication cycles through multiple mechanisms, including destabilization of viral envelopes, inhibition of cellular entry and intracellular trafficking, interference with transcription and translation, and prevention of virion maturation (Lee et al., 2022). These mechanisms operate either directly on viral particles or indirectly by targeting host cell receptors and modulating immune responses, thereby enhancing antiviral defense and maintaining homeostasis (Wild et al., 1992; Mookherjee et al., 2020).

AVPs display broad-spectrum antiviral potential, destabilizing viral membranes, inhibiting DNA replication, damaging virions, and reducing infectivity (Ahmed et al., 2019; Pu et al., 2019). Against non-enveloped viruses such as rhinoviruses (RVs), human papillomavirus (HPV), and adenoviruses (AVs), AVPs can block capsid uncoating and prevent nuclear entry of viral genomes (Chen et al., 2013; Lee et al., 2022). Certain AVPs, including those targeting herpes simplex virus (HSV) and human immunodeficiency virus (HIV), interact with host cell receptors essential for viral entry, while others, like CHDPs, exert immunomodulatory effects that enhance phagocytosis and regulate cytokine signaling (Morimoto et al., 1991; Mookherjee et al., 2020). This combination of direct virucidal and host-directed activities contributes to their high efficacy, low toxicity, and clinical potential (Liu et al., 2023).

Several AVPs have been translated into clinically approved drugs. Enfuvirtide (T-20) is the first FDA-approved fusion inhibitor for HIV, while boceprevir and telaprevir target the Hepatitis C Virus (HCV) nonstructural 3/4 protease, effectively blocking viral replication (Dando and Perry, 2003; Divyashree et al., 2020). AVPs have also shown promise against SARS-CoV-2, highlighting their potential for emerging viral threats (Chowdhury et al., 2020). Databases such as APD3, AVPdb, and ParaPep consolidate information on AVPs, facilitating the identification, design, and optimization of new antiviral peptides (Ourth, 2004; Wang et al., 2017). Collectively, AVPs represent a versatile and potent class of antiviral agents capable of addressing viral resistance and emerging infections while providing a framework for future drug development.

## Antifungal activity

Fungal infections range from common ailments such as fungal nail infections and thrush to life-threatening diseases like invasive candidiasis (Lee et al., 1999). The arsenal of effective antifungal agents remains limited, primarily comprising polyenes (amphotericin B), triazoles (fluconazole), echinocandins (caspofungin), and adjunctive drugs such as 5-flucytosine (De Lucca and Walsh, 1999). Amphotericin B, a polyene macrolide produced by *Streptomyces nodosus via* a polyketide synthase (PKS), is the only natural product with systemic antifungal activity (Buda et al., 2020). Widespread use of these drugs in healthcare and agriculture has contributed to the emergence of resistant strains of *Candida*, *Cryptococcus*, and *Aspergillus*, while some pathogens like mucorales and *Candida auris* exhibit intrinsic resistance (Fisher et al., 2018). Additionally, fungal biofilms, which form on both biotic and abiotic surfaces including medical devices, significantly enhance resistance and complicate treatment (Yang et al., 2022).

Antifungal peptides (AFPs) have shown promising activity against clinical, agricultural, and food-associated fungi, including *Aspergillus*, *Candida albicans*, and filamentous molds (Yang et al., 2022). They discriminate between mammalian and fungal cells by targeting fungal cell wall and plasma membrane components such as sphingolipids, chitin, β-glucans, and mannoproteins (Essig et al., 2014). AFPs exert antifungal effects through multiple mechanisms, including disruption of membrane integrity, interference with mitochondrial function as observed with histatin-5 and inhibition of intracellular processes (Mookherjee et al., 2020). For instance, AMP-17, derived from *Musca domestica*, demonstrates potent *in vitro* activity against both planktonic cells and biofilms of *Cryptococcus neoformans*, destabilizing membranes through multi-target interactions, thus offering a promising therapeutic approach for cryptococcal infections (Yang et al., 2022; Buda et al., 2020).

The scarcity of effective antifungal agents and the rising incidence of resistant strains underscore the urgent need to develop novel antifungal therapies. AFPs, with their ability to selectively target fungal cells, disrupt biofilms, and modulate intracellular pathways, present a valuable resource for addressing clinical and agricultural fungal challenges (Zhang and Zhu, 2009).

## Antiparasite activity

Neglected tropical diseases (NTDs) cause substantial mortality and morbidity, with approximately 200,000 deaths and 19 million disability-adjusted life years (DALYs) lost annually, affecting over one billion people worldwide (Olano et al., 2011; Jones et al., 2008; WHO, 2014). Diseases such as lymphatic filariasis, onchocerciasis, schistosomiasis, trypanosomiasis, leishmaniasis, and malaria caused by *Plasmodium* spp. pose serious health, social, and economic challenges (Shahabuddin et al., 1998; Flannery et al., 2013). The rise in drug-resistant parasites has created an urgent need for novel therapeutic strategies. Antiparasitic peptides (APs), a subset of AMPs, have demonstrated efficacy against pathogens responsible for malaria, leishmaniasis, and trypanosomiasis (ECDC, 2014; Pitale et al., 2020). For instance, Palestine-2 from honeybee venom showed significant anti-leishmanial activity without hemolytic effects on mouse macrophages or human erythrocytes (Pitale et al., 2020).

Insects, as vectors for many parasitic diseases, produce diverse AMPs, including magainins, defensins, cecropins, and melittin, which exhibit strong antiparasitic activity (Torrent et al., 2012). Apidaecin, an 18–20 residue peptide from *Apis mellifera*, displays both antibacterial and antiparasitic activity with minimal cytotoxicity (Li et al., 2006; Fieck et al., 2010). Synergistic combinations of insect-derived AMPs, such as magainin II, cecropin, and melittin, enhance antiparasitic potency, achieving IC50 values as low as 0.70 μM (Fieck et al., 2010). Melittin, a 26-residue peptide from honeybee venom, exhibits broad antimicrobial activity and inhibits kinases such as protein kinase C, protein kinase II, and myosin kinase, highlighting its potential for therapeutic applications beyond parasitic infections, including HIV, cancer, and neurological disorders (Verma et al., 2013). Its lethal dose (LD100) against trypomastigotes has been reported as 30 μM, emphasizing its potent antiparasitic effect. Overall, AMPs offer a promising avenue for treating parasitic diseases and NTDs, providing both direct antimicrobial effects and potential synergistic activity, which could help address the growing challenge of drug-resistant parasites.

## AMPs immune modulation

Natural peptides (NPs) exhibit a remarkable range of biological activities despite diverse sequences and structures. They are crucial effectors of innate immunity in both invertebrates and vertebrates, protecting against pathogens and modulating host defenses by disrupting microbial membranes, blocking vital cellular processes, influencing cytokine production, and activating downstream immune pathways that coordinate innate and adaptive responses (Boman, 2003; Lemire et al., 2013; Lyu et al., 2023). AMPs interact with the host immune system to modulate inflammatory responses, including acting as chemoattractants and activating classical complement pathways (Gan et al., 2021).

In chronic autoimmune diseases such as systemic lupus erythematosus and scleroderma, cytokine-induced cathelicidin expression and overexpression of human defensins confer protection against skin infections (Hilchie et al., 2013). AMPs recruit and activate immune cells during infection, enhancing microbial killing and controlling inflammation in both *in vitro* and *in vivo* models, including sepsis (Mookherjee et al., 2020). Their anti-inflammatory properties can suppress both acute and chronic inflammatory conditions (Frasca and Lande, 2012). Synthetic innate defense regulators (IDRs), such as IDR-1 and IDR-1018, mimic natural AMPs and suppress pro-inflammatory cytokines through adaptive immune pathways involving T and B cells (Kumar et al., 2018).

For example, IDR-1018 has been shown to reduce harmful neural inflammation in severe malaria when used alongside anti-malaria agents, without exhibiting direct anti-parasitic activity (Nijnik et al., 2010). Understanding the mechanisms by which CHDPs modulate immunity is crucial for developing peptide-based therapeutics that protect against infection, resolve inflammation, and maintain immune homeostasis (Powers and Hancock, 2003). CHDPs are also linked to alterations in the host gut microbiota, regulating microbial community composition and enhancing defense against pathogens (De Smet and Contreras, 2005). These multifaceted effects underscore the importance of multidisciplinary research to harness AMPs for the development of novel anti-infective and immunomodulatory therapies.

## Resistance mechanisms of AMPs

**Figure 3:**
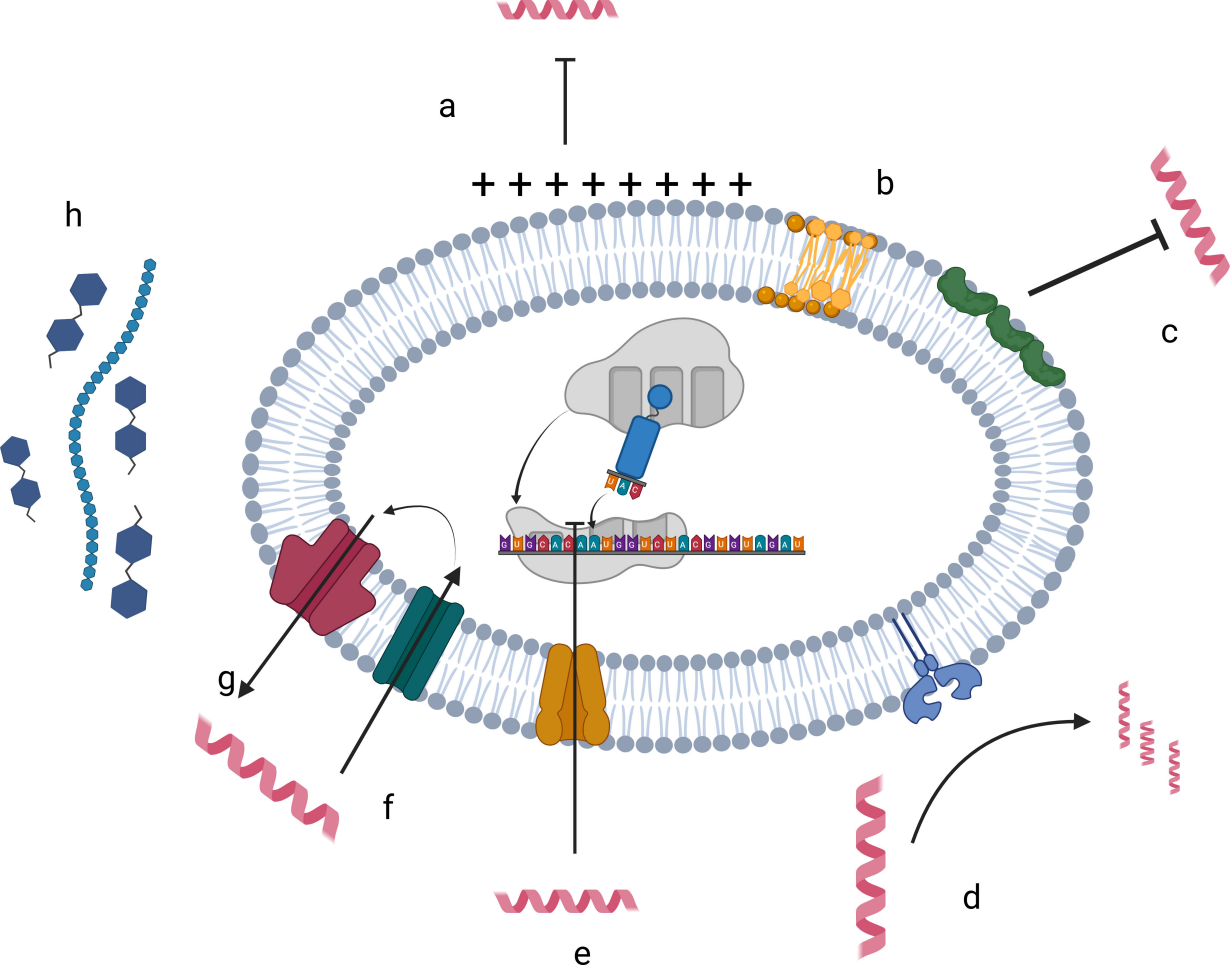
Mechanisms of antimicrobial resistance. AMP resistance mechanisms are changes in **(a, b)** cell envelope charge and composition modification, **(c)** producing surface or secreted proteins that bind and sequester AMPs, **(d)** degradation of AMP by proteases, **(e)** switching on and off of gene expression, **(f, g)** extrusion by efflux pumps and transport systems, and **(h)** released capsular polysaccharide. (Created in BioRender. Ngashangva, N. (2025) https://BioRender.com/sdl7tq9).

AMP cross-resistance through diverse structural and functional mechanisms could limit their utility as microbicides (Maria-Neto et al., 2015). Prolonged exposure to therapeutic AMPs may induce resistance, reducing effectiveness against both naturally occurring host AMPs and administered peptides (Bechinger and Gorr, 2017; Mader and Hoskin, 2006; Afacan et al., 2012). Band and Weiss outlined four primary resistance strategies: (1) proteolytic degradation of peptides, (2) bacterial surface modifications that alter AMP targets, (3) membrane shielding *via* capsule production, and (4) downregulation of AMP activity through immunomodulation. In effect, bacteria destroy AMPs, alter their targets, shield themselves, or interfere with host immune responses (Band and Weiss, 2015) (**Figure 3**).

A major resistance pathway involves the PhoP/PhoQ two-component regulatory system, which modulates transcriptional responses to AMP stress and nutrient limitation. AMPs activate PhoQ kinase, which transfers a phosphate to PhoP, regulating AMP resistance genes (Bader et al., 2005). This system is present in GNB such as *Shigella flexneri*, *Y. pestis*, and *P. aeruginosa*, as well as in GPB like *Streptomyces* spp., facilitating peptide expulsion or inactivation *via* proteolytic degradation (Li et al., 2007). In GPB, secreted proteases neutralize AMPs extracellularly, whereas in GNB, proteases are often membrane-bound. For example, aureolysin, a metalloprotease from *Staphylococcus aureus*, hydrolyzes and inactivating the C-terminal domain of AMPs like LL-37 (Nawrocki et al., 2014).

Bacteria also evade AMPs by modifying membrane charge and composition. Negatively charged lipids, such as phosphatidylglycerol (PG) and cardiolipin, can be replaced or modified by covalent attachment of Lys or alanine (Ala) residues, reducing AMP binding (Andersson et al., 2016; Bechinger and Gorr, 2017). D-alanylation *via* the dlt operon, lysinylation, O-acetylation, N-deacetylation of peptidoglycan, and glycosylation of wall teichoic acids collectively decrease membrane negativity, protecting against AMPs including human LL-37 and Cathelicidin-related antimicrobial peptide (CRAMP) in mice (Abi Khattar et al., 2009; Aubry et al., 2011; Meireles et al., 2020).

Secreted or surface-bound proteins also confer resistance by sequestering AMPs. *Streptococcus pyogenes* secretes Streptococcal Inhibitor of Complement (SIC) and M protein serotype 1 (M1), both of which inactivate LL-37, enhancing bacterial invasiveness (Nawrocki et al., 2014). Additionally, bacteria employ efflux pumps and ABC transporters to actively remove AMPs from the cell (Collins et al., 2010). These include adenosine triphosphate (ATP) driven primary transporters and secondary transporters driven by electrochemical gradients, with examples such as RosA/RosB in *Yersinia enterocolitica*, MtrCDE in *Neisseria gonorrhoeae*, and SpaIFEG in *Bacillus subtilis* to prevent self-damage from secreted AMPs (Stein et al., 2005; Gaurav et al., 2023). Resistance is further modulated by cell wall signaling through sigma factors and global regulators (σB, σL, VirR, LiaR) controlling genes like *dlt*, *mpr*F, and ABC transporters, enhancing bacterial survival under AMP stress (Bergholz et al., 2013).

Biofilms represent another critical barrier. The extracellular slime matrix, primarily composed of exopolysaccharides, acts as both a physical and chemical shield, repelling AMPs and modifying their structure, reducing efficacy (Magana et al., 2020). For example, *P. aeruginosa* produces alginate, while GPB like *S. aureus* use polysaccharides such as poly-N-acetylglucosamine (PIA); deacetylation of PIA further enhances biofilm resistance (Band and Weiss, 2015).

Although high-level AMP resistance may emerge, it is generally slower than resistance to conventional antibiotics and depends on administration methods. Careful design and delivery of AMPs combined with antibiotic or peptide co-therapy can reduce resistance development and attenuate pathogen virulence (Sanguinetti et al., 2015; Matejuk et al., 2010). These multifaceted strategies underscore the dynamic nature of bacterial defenses and the challenges in employing AMPs therapeutically, highlighting the need for continued research and innovative approaches to circumvent resistance.

## Connecting chemotype to genotype

Microbial extracellular SMs play key roles in microbe-host and microbe-microbe interactions, with many organisms producing SMs that possess antimicrobial activity and influence ecological functions (Rosenblueth and Martinez-Romero, 2006; Klaenhammer, 1988). However, numerous novel biomolecules remain undiscovered due to technological limitations and research biases toward known compounds. Linking these diverse SMs, including AMPs, to their biosynthetic genes *via* genomics, proteomics, and metabolomics provides a powerful strategy for discovering novel molecules (Cimermancic et al., 2014; Nijman, 2015). Tools such as AntiSMASH identify and annotate biosynthetic gene clusters (BGCs) in microbial genomes, whereas Joint Genome Institute secondary metabolism collaboratory (JGI SMC) enables large-scale comparison and analysis across species. AntiSMASH excels in detecting individual BGCs but is constrained by incomplete references and assemblies, while JGI SMC offers broader insights dependent on the quality of input BGC data (Blin et al., 2025; Udwary et al., 2025).

The Nature Product Peptidogenomics (NPP) method uses mass spectrometry (MS)-guided chemotype-to-genotype mining to iteratively match *de novo* tandem MS/MS data with corresponding BGCs in sequenced organisms, efficiently identifying novel peptides and their biosynthetic genes (Challis, 2008; Srinivas et al., 2010; Mohammad et al., 2015; Hancock and Chapple, 1999; Madsen et al., 2022). For example, NPP characterized the ribosomal lantipeptide AmfS from *Streptomyces griseus* IFO 13350 and the non-ribosomal lipopeptide stendomycin I from *Streptomyces hygroscopicus* ATCC 53653 along with their respective BGCs (Kersten et al., 2011). Similarly, transcriptomic and bioinformatic analysis of *Bacillus halodurans* C-125 revealed genes encoding precursor peptides clustered with LanM-type synthetases. MS analysis of cell-free supernatants (CFS), *in vitro* enzyme reconstitution, antimicrobial assays, and mutagenesis enabled structural characterization of the two-component lantibiotic haloduracin (Velasquez and van der Donk, 2011).

**Figure 4:**
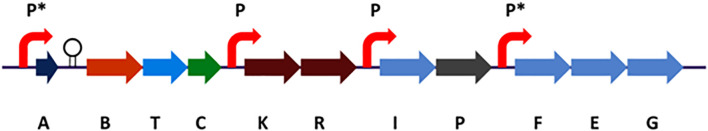
Schematic illustration of the transcriptional organization of nisin biosynthetic gene cluster adapted from Lubelski et al., 2008. The genes for nisin biosynthesis are transcriptionally organized into four operons, i.e., *nis*ABTC, *nis*IP, *nis*RK, and *nis*FEG. The ribosomally synthesized precursor nisin (Nis A) is encoded by the *nis*A gene. Specific Ser and threonines (Thr) in the core peptide of precursor nisin are intracellularly dehydrated by the dehydratase *Nis*B; subsequently, the dehydrated amino acids are added by the cyclase *Nis*C, forming thioether ring-like structures (Lubelski et al., 2008). The fully modified precursor nisin is exported to the exterior by the ABC transporter *Nis*T, followed by the cleavage of the leader peptide by the extracellularly located Ser protease *Nis*P to release active nisin. *nis*I gene is preceded by an internal promoter, which enables the expression of the immunity protein before full nisin production is established. The regulatory two-component system (TCS) genes, *nis*RK, are preceded by a weak promoter. The fourth operon encodes the immunity proteins nisFEG. Promoters marked P* are controlled by a TCS NisRK, whereas transcription of nisRK and nisI (P) is constitutive (Lubelski et al., 2008).

BGCs often contain accessory genes, including transporters, regulatory elements, and immunity genes, in addition to core biosynthetic enzymes, which contribute to peptide assembly, maturation, and host resistance (Barra et al., 1998; Walsh and Fischbach, 2010). *In silico* mining of the human microbiome using metagenomic libraries from the Human Microbiome Project (HMP) and profile hidden Markov models (HMMs) has revealed bacteriocin gene clusters across diverse body niches, including the gut microbiome (Walsh et al., 2017; Chu et al., 2016). Genes for nisin production in *Lactococcus lactis*, encompassing maturation, immunity, and regulation, are located on the conjugative transposon Tn5276, which also carries determinants of sucrose metabolism (**Figure 4**). The biosynthesis of nisin and its small-molecule products has been reconstituted in heterologous hosts (Lubelski et al., 2008; Chen et al., 2020; Xiang et al., 2009; Siegers et al., 1996).

In animal systems, full-length cDNA sequences of piscidin and hepcidin AMPs from *Seriola lalandi* encode a 65-residue prepropeptide, including a 25-residue mature peptide predicted to form an amphipathic helix-loop-helix structure (Muncaster et al., 2018; Diamond et al., 1991). We illustrated the 3-D conformation of an NRPS predicted from LC-MS/MS analysis of *Paenibacillus peoriae* IBSD35 (PTJM00000000) metabolomics, deposited at ModelArchive (A0A2S6P0H9; ma-z4hip) (Ngashangva et al., 2021). Integrating genomics, metabolomics, genome mining, and structural elucidation enhances the discovery of novel AMPs that are stable, specific, and effective in countering AMR (Ngashangva et al., 2022).

## Therapeutic applications of peptides

Insulin was the first commercial peptide drug, introduced in 1923, offering life-saving therapy for diabetes (Vecchio et al., 2018; Wang et al., 2022). Decades later, peptide-based antimicrobials gained prominence: gramicidin and polymyxin B were included in Neosporin^®^ in 1955, followed by colistin (polymyxin E) in 1962, and daptomycin in 2003 to counter antibiotic-resistant bacteria (Chen and Lu, 2020). Nisin, a 34-amino-acid AMP with antimicrobial activity against GPB, has been successfully applied in pharmaceuticals and food preservation to treat colonic infections (McAuliffe et al., 2001). Currently, several AMPs, including nisin, gramicidin, polymyxins, daptomycin, vancomycin, oritavancin, dalbavancin, telavancin, and melittin, are in clinical use as alternatives to conventional antibiotics. Since 2000, numerous peptides such as setmelanotide, edotreotide gallium Ga-68, lutetium Lu 177 dotatate, etelcalcetide, afamelanotide, lixisenatide, carfilzomib, tesamorelin, mifamurtide, icatibant, pramlintide, ziconotide, enfuvirtide, teriparatide, carbetocin, taltirelin, and nisin A have been FDA-approved (**Table 2**) (Chen and Lu, 2020). Additionally, lucinatant, linaclotide, peginesatide, dulaglutide, CB183315, and oritavancin are either on the market or in development (Kaspar and Reichert et al., 2013). Over 60 peptide drugs are FDA-approved, with more than 400 under clinical evaluation (Wang et al., 2022).

**Table 2:** Peptide-based drugs approved since 2000 along with their targets and indications for approval.

Target name	Peptide name	Year of approval	Approved indication
Melanocortin-4 receptor	Setmelanotide	2020	Indicated for chronic weight management of obesity
Somatostatin receptors	Edotreotide gallium Ga-68	2019	Indicated for diagnose somatostatin receptor positive neuroendocrine tumors
Somatostatin receptors	Lutetium Lu 177 dotatate	2018	Treatment of somatostatin receptor-positive gastroenteropancreatic neuroendocrine tumors
AT1 receptor	Angiotensin II 483	2017	Indicated for sepsis and septic Shock
CaSR	Etelcalcetide	2016	Indicated for secondary hyperparathyroidism
MC1 receptor	Afamelanotide	2014	Prevention of phototoxicity
GLP-1 receptor	Lixisenatide	2013	Indicated for Type 2 Diabetes Mellitus
Binding to active site of the 20S proteasome	Carfilzomib	2012	Treatment of multiple myeloma
GHRH receptor	Tesamorelin486	2010	Reduction of HIV lipodystrophy
NOD2 protein	Mifamurtide474	2009	Treatment of high-grade, resectable, non-metastatic osteosarcoma
Beta2-receptor	Icatibant	2008	Approved for use in acute attacks of hereditary angioedema
Calcitonin receptor	Pramlintide	2005	Treatment of Type 1 and Type 2 Diabetes Mellitus
N-type calcium channels	Ziconotide	2004	Management of severe chronic pain
gp41	Enfuvirtide	2003	Used in combination therapy for the treatment of HIV-1
PTH1 receptor	Teriparatide	2002	Treatment of osteoporosis
OT receptor	Carbetocin	2001	Used for postpartum hemorrhage
TRH receptor	Taltirelin	2000	Spinocerebellar degeneration
Nisin	Nisin A	1988	A food preservative in 1988 due to its heat stability and tolerance of low pH

AMPs in preclinical and clinical studies, including histatin, plectasin, omiganan, IMX942, iseganan, LL-37, and P113, are being explored for anti-infective applications (**Table 3**). Combination therapy using AMPs with conventional antibiotics or other peptides has shown additive or synergistic effects. For example, AMP R9 conjugated to magainin-M15 enhanced translocation across lipid bilayers and increased antimicrobial activity against GNB, while nisin combined with penicillin or chloramphenicol improved efficacy against *Enterococcus faecalis* (Lee et al., 2019; Tong et al., 2014).

**Table 3:** Peptide-based drugs currently available on the market and in the development pipeline.

Code name	Mechanism of action	Disease treatment
Lucinatant	Lung surfactant	Respiratory distress syndrome
Linaclotide	Receptor agonist	Gastrointestinal disorders
Peginesatide	Receptor agonist	Anemia, Chronic kidney disease
Carfilzomib	Protease inhibitor	Hematological cancer
Dulaglutide	GLP-1R agonist	Type 2 diabetes mellitus
Oritavancin	GPB cell membrane disruption	Acute bacterial skin and skin structure infections
CB183315	*Clostridium difficile* cell membrane disruption	*C. difficile* associated Diarrhea

Beyond antibacterial activity, AMPs exhibit antiviral and anticancer properties. Cecropin A inhibits HIV replication by suppressing viral gene expression and is active against herpes simplex virus (HSV) types 1 and 2 and Junin virus (JV) (Vilas Boas et al., 2019). AMPs selectively target cancer cells due to the higher expression of negatively charged molecules, such as heparan sulfate, O-glycosylated mucins, and sialylated gangliosides, which increase membrane fluidity and transmembrane potential (Felicio et al., 2017). Aurein 1.2, for instance, demonstrates cytotoxicity against approximately 50 cancer cell lines with minimal effect on healthy cells (Rozek et al., 2000). This selective targeting involves binding to phosphatidylserine (PS) exposed on cancer cell membranes, leading to membrane disruption and cell death, presenting a promising alternative to conventional chemotherapy (Thundimadathil, 2012; Morgan et al., 2011).

## AMPs under clinical trials

AMPs hold significant promise as therapeutic agents for preventing and treating infections. However, their clinical translation remains limited due to challenges such as short half-life, poor bioavailability, susceptibility to enzymatic degradation, and high production costs, necessitating innovative delivery strategies (Muttenthaler et al., 2021; Asif et al., 2024; Stielow et al., 2023). Despite these barriers, several AMPs have advanced through clinical trials, demonstrating diverse mechanisms including membrane disruption, immunomodulation, and target-specific activity, highlighting their potential to address AMR and unmet therapeutic needs (**Table 4**).

**Table 4:** Peptide-based drugs in clinical trials that showcased and reinforced their therapeutic potential.

Name	Description	Medical use	Stage of development	Company
Nisin A (Type A lantibiotic)	*Lactococcus lactis* subsp	Urogenital tract infections; Spermicidal activity (Rabbit model; *In-vivo*)	Preclinical	Unknown
IDR-1	Derivative of bactenecin from bovine neutrophils	Prevention of infections in the immune compromised	Phase 1	Unknown
DP178 (T20, Enfuvirtide & Fuzeon)	C-terminus of the ectodomain of gp41	HIV-1 membrane fusion inhibitor; AIDS	Proved by FDA	Trimeris
Colicin E1 (Bacteriocin)	*E. coli* H22	Gastrointestinal tract infections; Stomach and intestine infections (Murine model; *In-vivo*)	Preclinical	Unknown
Bacteriocin OR-7	*Lactobacillus salivarius* NRRL B-30514	Gastrointestinal tract infections; Campylobacter infection (Chicken model; *In-vivo*)	Preclinical	Unknown
MX-594AN	Indolicidin based	Treatment of catheter-related acne	Phase IIb Completed	Migenix (Vancouver, British Columbia, Canada)
HB-107	19-amino-acid fragment of cecropin B	Wound healing	Preclinical	Helix Biomedix (Bothell, Washington, USA)
PTX001	Potent anti-angiogenic agents	Broad-spectrum antimicrobial; Antiendotoxin	Preclinical	PepTx
Locilex	22-amino acid magainins	Infections of diabetic foot ulcers	Phase III (Failure)	Genaera
Murepavadin	POL7080, a novel 14–amino acid cyclic β-hairpin peptide	Cystic fibrosis	Phase III (Failure)	Polyphor

Nisin, widely approved as a food preservative in over 80 countries, is also being evaluated for treating oral cavity squamous cell carcinoma and exhibits spermicidal activity *in vivo* without causing inflammation or tissue damage (Chikindas et al., 2018; Reddy et al., 2004). Hlf1-11, derived from human lactoferrin, shows *in vivo* efficacy against MRSA, *Klebsiella pneumoniae*, and fungal pathogens, with additional immunomodulatory effects, including promotion of monocyte differentiation and pro-inflammatory cytokine release (van der Does et al., 2012). The specifically targeted AMP (STAMP) C16G2 is in phase II trials for preventing dental caries by selectively targeting *Streptococcus mutans* while preserving the oral microbiome (Kaplan et al., 2011; Sullivan et al., 2011).

Synthetic and derivative peptides have also demonstrated therapeutic potential. 1DR-1018, a derivative of bovine cathelicidin bactenecin, promotes wound healing by enhancing keratinocyte migration and proliferation at doses much lower than LL-37, with reduced cytotoxicity (Steinstraesser et al., 2012). Fuzeon, a synthetic fusion inhibitor, blocks HIV-1 gp41-mediated viral entry and is used in combination therapy for HIV/AIDS (Qiu et al., 2012). Omiganan (MBI-226), an indolicin-based AMP, completed phase III trials for catheter-related infections, demonstrating safety and efficacy against both GPB and GNB (Fjell et al., 2012; Friedrich et al., 2000).

Emerging AMPs with unique mechanisms include dusquetide (SGX942), a first-in-class IDR that modulates immune responses *via* the p62 adaptor protein, currently in phase III trials for oral mucositis (Kudrimoti et al., 2016), and ramoplanin (NTI-851), which disrupts GPB cell wall synthesis, in phase III trials for vancomycin-resistant *Enterococcus* and phase II trials for *C. difficile* infections (Alshrari et al., 2023). Novexatin^®^ (NP213), an AFP, is being evaluated for onychomycosis, functioning by penetrating the nail and disrupting fungal membranes without systemic exposure or significant adverse effects (Mercer et al., 2020).

Murepavadin (POL7080), a 14-amino acid cyclic β-hairpin peptide, targets the outer membrane protein LptD of *P. aeruginosa*, disrupting LPS assembly and causing cell death. It has demonstrated potent activity against extensively drug-resistant (XDR) strains, including colistin-resistant isolates, and is being developed as an inhaled therapy for cystic fibrosis. However, its intravenous phase III trials for nosocomial pneumonia/ventilator-associated bacterial pneumonia (HABP/VABP) were halted due to a high incidence of acute kidney injury (nephrotoxicity) (Martin-Loeches et al., 2018).

Locilex (pexiganan 0.8% cream), a 22-amino-acid synthetic analogue of magainin II, was developed for treating mild diabetic foot ulcer infections. However, it failed to meet both primary and secondary endpoints: it was not superior to vehicle plus standard of care in achieving clinical success, nor did it demonstrate improved bacterial eradication. Additionally, higher-than-expected serious adverse events including increased rates of osteomyelitis and cellulitis were reported (Da Silva et al., 2021).

Recent advances in AMPs have highlighted their potential as therapeutic agents against infections and cancer. A Phase I/II trial completed in 2024 evaluated an LL-37-derived peptide for melanoma, demonstrating safety and tolerability *via* intratumoral injections and revealing modulation of the tumor microenvironment that may enhance immune responses against tumor cells (Zheng et al., 2025). In antimicrobial applications, 58 venom-derived peptides (VEPs) were identified and synthesized, showing potent activity against multiple WHO-priority bacterial pathogens through computational discovery and experimental validation (Del Olmo and Andreu, 2025). Among these, Mastoparan X from wasp venom displayed strong bactericidal activity against MRSA, including the USA300 strain, by disrupting bacterial membranes and reducing biofilm formation, indicating its promise as a novel anti-MRSA agent (Lu et al., 2025). These studies collectively underscore the versatility of AMPs in targeting MDR pathogens and in repurposing existing peptides for oncology, illustrating the growing translational potential of AMPs as next-generation therapeutics.

Despite these promising candidates, the clinical advancement of AMPs is challenged by formulation difficulties, susceptibility to proteolytic degradation, potential inflammatory effects, and production costs. Nonetheless, advances in peptide design, delivery systems, and combination therapies are progressively addressing these limitations, reinforcing AMPs as a transformative avenue for combating AMR and unmet infectious disease needs.

## Limitations of AMPs

AMR is an ancient, natural adaptation of microbes, arising through mutations, gene amplification, and horizontal gene transfer (Peschel and Sahl, 2006; DCosta et al., 2011). Resistance genes have even been detected in pristine environments with no prior antimicrobial exposure, showing that bacteria evolved such traits long before human drug production (Larsson and Flach, 2022). It is reported that AMPs, like conventional antibiotics, encounter natural resistance mechanisms in diverse environments (Band and Weiss, 2015; Andersson et al., 2016).

Bacteria resist AMPs through multiple strategies, including altering outer membrane composition to reduce peptide binding, secreting proteases that degrade peptides, and forming biofilms that block AMP penetration (Zasloff, 2002; WHO, 2022). Sub-inhibitory concentrations of polymyxin B and colistin can even promote biofilm formation, further complicating therapeutic use (Sato et al., 2018). These resistance traits are also present in host-associated microbiota, where bacteria can tolerate endogenous host-defense peptides, posing a challenge to AMP-based drug design (Ostaff et al., 2013).

In addition to microbial resistance, AMPs face pharmacological limitations. They often show cytotoxicity, antigenicity, and allergic potential, with variable safety profiles in mammalian systems (Zhang et al., 2021; Oman and van der Donk, 2010; Fox, 2013). Their pharmacokinetic properties are also unfavorable: peptides are rapidly degraded by proteolytic enzymes, exhibit short plasma half-lives (often <30 minutes), and have poor oral bioavailability due to instability at acidic pH and low intestinal absorption (Diao and Meibohm, 2013; Azman et al., 2022). These features lead to limited systemic exposure and reduced efficacy at the infection site.

Despite their theoretical advantages, AMPs have not yet demonstrated consistent superiority over conventional antibiotics in phase II or III clinical trials (Elad et al., 2012). Many therapeutic peptides fail *in vivo* due to toxicity, including hemolysis from membrane disruption and nephrotoxicity from renal accumulation. Selectivity seen *in vitro* collapses in physiological environments with serum proteins and diverse membranes, while rapid proteolysis and renal clearance cause poor pharmacokinetics and limiting their efficacy (Wang et al., 2021, Wang et al., 2022). Combined with high production costs and formulation challenges, these barriers currently prevent AMPs from becoming widely adopted therapeutic alternatives.

## Cost barriers in production and peptide manufacturing

Extracting natural AMPs remains limited by low yield, impurities, labor-intensive protocols, and high costs associated with methods. To overcome these challenges, scalable alternatives like chemical synthesis and recombinant production are being explored (Fang et al., 2024). Bioactive peptides can be obtained *via* biosynthesis (enzymatic hydrolysis, microbial fermentation, recombinant DNA technology) or chemosynthesis (solution- and solid-phase methods). Their purification relies on membrane separation, chromatography, or electrophoresis, while structural identification employs mass spectrometry and NMR (Reimann et al., 2019). Peptide nanostructures are stabilized by non-covalent forces, including hydrogen bonding, hydrophobic interactions, and π-π stacking (Wen et al., 2023).

Traditional methods, such as ammonium sulfate precipitation from CFS, remain time-consuming and yield impure products (Indira et al., 2022). Recombinant DNA technology has transformed AMP production, though translation into clinical use is constrained by costly high-throughput synthesis and the time required for screening (Cesaro et al., 2023).

Solid-phase peptide synthesis (SPPS), first described by Merrifield in 1963, revolutionized chemical production by coupling and deprotecting amino acids on a solid resin support (Merrifield, 1963). Modern advances include automated synthesizers and peptide libraries, which facilitate homogeneous peptide preparation at laboratory scale and have greatly accelerated therapeutic peptide development (Wang et al., 2022; Sadeeq et al., 2025). Recombinant systems offer another major route. Prokaryotes such as *E. coli* are widely used because of their low cost and high yield (Musa et al., 2016). However, they often lack the ability to perform PTMs. Eukaryotic hosts, such as yeast (*Saccharomyces cerevisiae*, *Pichia pastoris*), allow glycosylation, signal peptide processing, and disulfide bond formation, which are critical for Cys-rich AMPs (Mattanovich et al., 2012; Cereghino and Cregg, 2000). Successful expression of cecropins, defensins, ABP-CM4, CAP18/LL37, and hybrid peptides has been demonstrated in *P. pastoris* (Wang et al., 2011; Hsu et al., 2009). Despite higher production costs, yeast systems are advantageous for producing complex AMPs.

Plant-based bioreactors represent a promising low-cost alternative, requiring only soil, water, and light. Transgenic strategies have enabled AMP gene expression in crops such as corn, soybean, and tobacco (Shanmugaraj et al., 2021). High yields of AMPs like Cn-AMP1, clavanin A, Cm-AMP5, and parigidina-br1 were achieved in tobacco leaves (Leite et al., 2019). Recent advances demonstrated efficient production of recombinant and amidated AMPs in *Nicotiana benthamiana*, including the clinical peptide IDR-1002, through transient expression systems that reduce plant toxicity and downstream processing costs (da Cunha et al., 2017; Chaudhary and Mahfouz, 2024). Chemical synthesis, microbial fermentation, and plant-based systems have all advanced AMP production, scalability remains hindered by cost, stability, and efficiency challenges. Continued innovation in expression systems and downstream processing is essential to make AMP manufacturing commercially viable.

Recent innovations are addressing longstanding cost barriers. Cell-free protein synthesis (CFPS) platforms allow AMP production without living hosts, avoiding issues of peptide toxicity to microbial cells, enabling rapid prototyping, and supporting the generation of diverse peptide libraries at reduced cost (Lee et al., 2023). Similarly, microfluidic high-throughput screening integrates peptide synthesis, folding, and antimicrobial activity testing in miniaturized systems, lowering reagent costs while enabling large-scale functional screening (Ma et al., 2022; Devrani et al., 2025). These approaches, when combined with ML-guided design, hold promise for accelerating the discovery and scalable production of next-generation AMPs with reduced manufacturing costs.

Despite rapid advances in AMP discovery, major non-scientific barriers impede commercialization. Regulatory agencies such as the FDA and European Medicines Agency (EMA) lack clear approval pathways for peptide therapeutics, resulting in additional requirements for toxicity, immunogenicity, and manufacturing consistency (Elsayed et al., 2025). Patentability further limits translation, natural peptides are difficult to protect because they may be considered products of nature, and strong patents are typically granted only for synthetic modifications (Domingo-Fernandez et al., 2024). Economically, AMPs suffer from the antibiotic market failure: high development cost and low return discourage investment without incentives such as market entry rewards or subscription reimbursement models (Theuretzbacher et al., 2020).

## Rediscovering AMPs

Advanced strategies are crucial to optimize AMPs by improving potency, stability, bioavailability, delivery, immunomodulatory activity, and lowering toxicity and production costs (Melo et al., 2006; Lam, 2007; Gautam et al., 2016). Conventional methods include amino acid substitution, gene overexpression, cloning, peptide coating, hydrophobic end-tags, epitope mapping, and pharmacophore-based modifications (Pasupuleti et al., 2009). More recent approaches emphasize peptidomimetics, AI-guided design, and advanced drug delivery platforms (Ho et al., 2024).

Xiao et al. highlight several formulation strategies that are advancing peptide-based therapeutics. Terminal modifications, particularly N-acetylation, C-amidation, and albumin fusion, enhance proteolytic stability and are key approaches for prolonging plasma half-life. Beyond D-amino acid substitution, they identify backbone cyclisation, side-chain modification, and incorporation of non-natural amino acids as major structural design principles to improve stability and activity (Xiao et al., 2025). Despite these advances, achieving oral or alternative delivery routes with sufficient stability and bioavailability remains a significant challenge in peptide drug development.

Nanotechnology enhances AMP performance by improving solubility, circulation time, biofilm penetration, and protection against enzymatic degradation (Bahrami et al., 2020; Carnero Canales et al., 2025). Nanostructures such as nanotubes, graphene, and metal nanoparticles facilitate targeted delivery; for instance, diphenylalanine nanotubes selectively eradicated Gram-positive biofilms while reducing host toxicity (Carratalá et al., 2020). These nanosystems also improve oral bioavailability (Roque-Borda et al., 2022). In parallel, structure-based drug discovery including protein protein interaction (PPI)-guided peptide design offers complementary strategies (Wang et al., 2022).

Chemical modifications such as cyclization, phosphorylation, acetylation, halogenation, and incorporation of D- or unnatural amino acids enhance protease resistance, selectivity, and pharmacokinetics (Zhong et al., 2020; White and Yudin, 2011). Charge engineering and analog development further expand antimicrobial and anti-inflammatory properties (Timón et al., 2019; Kong et al., 2022). Genetic and biosynthetic tools, including NRPS engineering, site-directed mutagenesis, and heterologous expression, support high-throughput creation of novel AMP variants (Xiang et al., 2009; Yan et al., 2018).

Biophysical modeling, including molecular dynamics, free energy perturbation, and membrane interaction simulations, guides AMP optimization to maximize antibacterial activity while minimizing host toxicity (Torcato et al., 2012). Other promising directions include targeting bacterial-specific pathways, improving pharmacokinetic parameters, and designing synergistic combinations to slow resistance development (Cardoso et al., 2021; Gupta et al., 2019). Understanding resistance mechanisms remains critical for translation into clinical use (Maria-Neto et al., 2015).

The integration of high-throughput sequencing and AI is rapidly transforming AMP discovery to combat AMR. Sequencing technologies unlock vast genomic, proteomic, and metagenomic datasets, revealing an immense peptide sequence space that would be impossible to evaluate experimentally. AI models then processes this landscape with precision: discriminative models predict antimicrobial activity, toxicity, and stability, enabling efficient prioritization, while generative models learn sequence-activity patterns and design entirely novel peptides that may surpass natural AMPs (Szymczak et al., 2023). AI further enhances the pipeline through SARs forecasting and rapid navigation of large sequence libraries (Wan et al., 2024). This capability has already produced AMPs with potent antibacterial and antifungal efficacy *in vivo* (Wang et al., 2025), as well as small molecules active against *A. baumannii* (Swanson et al., 2024).

Sequencing-driven computational mining reinforces this progress by evaluating physicochemical properties such as net charge, hydrophobicity, and peptide length, Torres et al. scanned 42,361 human protein sequences and identified 2,603 peptide candidates, many previously unrecognized as antimicrobials, several of which were experimentally validated (Pane et al., 2017; Torres et al., 2024). Beyond the human proteome, large-scale mining of 63,410 metagenomes and 87,920 microbial genomes predicted nearly one million AMP candidates, compiled into the AMPSphere database (Santos-Júnior et al., 2024). Together, these advances demonstrate how sequencing provides unprecedented access to natural peptide diversity, while AI enables the design of realistic and idealized synthetic peptides, accelerating discovery and reducing the cost and uncertainty of traditional antibiotic development.

ML and bioinformatics accelerate discovery by predicting structure-activity relationships (SARs), optimizing charge, hydrophobicity, and amphipathicity, and avoiding dereplication of known peptides (Lee et al., 2016; Wang, 2015; Ciulla and Gelain, 2023). Display technologies (phage, yeast, mRNA, ribosome, DNA) have yielded macrocyclic ligands and therapeutic leads, advancing peptide-based drug pipelines (Josephson et al., 2014; Passioura et al., 2014; Zhang et al., 2014). Proteomics combined with deep learning-assisted mining has uncovered thousands of new AMPs, many showing *in vivo* efficacy against MDR and ESKAPEE pathogens (Witek et al., 2017; Ho et al., 2024).

AMPs serve as natural templates for designing novel therapeutics, and SAR studies are essential for linking chemical structure to biological activity, including antimicrobial potency, selectivity, and toxicity, specially the natural peptides templates where the function are strongly influence by the structure (**Supplementary Table S2**). Tang et al. developed ultrashort “tadpole-like” AMPs, comprising an amphipathic α-helical head fused to a flexible aromatic tail. Systematic variation of head charge, tail aromaticity, and peptide topology yielded HT2 and RI-HT2, which exhibited broad-spectrum activity, minimal cytotoxicity, and low resistance induction. SARs analysis revealed that increased head charge enhances electrostatic interactions with bacterial membranes, while the aromatic tail supports membrane insertion and stability, highlighting the importance of balanced charge and aromatic density (Tang et al., 2023).

Brunicardi et al. examined clovibactin using ala/D-residue scans and analogue profiling, identifying residues Phe¹, D-Leu², Ser_4_, D-Hyn_5_, Leu_7_, and especially Leu^8^ as critical for activity. Backbone integrity and stereochemistry were essential: N-methylation abolished activity, and the enantiomer was inactive. Clovibactin showed potent activity against GPB but negligible effects on GNB due to outer membrane barriers. SAR analysis indicated that hydrophobic residues drive binding to lipid II and other pyrophosphate-containing targets, while polar residues stabilize interactions, explaining its Gram-positive selectivity (Brunicardi et al., 2024).

Hernández-Ortiz et al. studied DICAMs, tripeptides conjugated to a fatty amine with net charges from −2 to +1. Activity depended on the hydrophobic tail, central Pro residue, and C-terminal Glu. A long aliphatic chain (C16–C18) and Pro were indispensable for membrane insertion and conformation, while the C-terminal Glu tolerated minor variations. N-terminal modifications modulated activity without abolishing it, and cationic charge was not required. DICAMs exhibited selective Gram-positive activity, with Gram-negatives resistant due to outer-membrane exclusion (Hernández-Ortiz et al., 2024).

Radzishevsky et al. investigated acyl-lysine oligomers, showing that long hydrophobic acyl chains (C12–C16) and di- or tri-lysine repeats were critical for potent antibacterial activity. Excessively long oligomers increased hemolysis, emphasizing the need to balance hydrophobic tails and cationic residues. Active oligomers adopted amphipathic conformations enabling membrane insertion, demonstrating that fine-tuning chain length and lys repeats can achieve broad-spectrum potency with minimal host toxicity (Radzishevsky et al., 2008).

Overall, these SAR studies collectively highlight that antimicrobial potency and selectivity rely on a precise balance of hydrophobicity, charge distribution, peptide conformation, and specific side-chain interactions. While hydrophobic anchoring and amphipathic structures are crucial for membrane disruption, stereochemistry, backbone integrity, and topology fine-tune activity and selectivity, often conferring higher potency against AMR pathogens.

## Conclusion

AMR remains one of the most pressing challenges in modern medicine, posing a profound threat to global health, food security, and economic stability. Driven by the natural evolution of microbes, horizontal gene transfer, environmental dissemination, and widespread misuse of conventional antibiotics, AMR compromises the effectiveness of existing therapeutic interventions and threatens to render previously treatable infections life-threatening. Conventional antibiotics, while historically transformative, are increasingly ineffective against MDR and XDR pathogens, highlighting the urgent need for innovative alternatives. In this context, AMPs have emerged as a promising class of bioactive molecules that offer broad-spectrum antimicrobial activity, immunomodulatory effects, antibiofilm properties, and a reduced likelihood of resistance development. The inherent versatility of AMPs, coupled with advances in chemical synthesis and modification, recombinant production, and AI-guided design, positions them as next-generation therapeutics.

The discovery and characterization of AMPs from diverse natural sources including bacteria, fungi, plants, amphibians, and insects have provided rich natural resources for the development of novel therapeutics. Both RSPs and NRPs exhibit remarkable structural diversity and PTMs, enhancing stability, antimicrobial potency, and functional versatility. RSPs, such as lantibiotics, are genetically encoded and can be expressed and modified with high specificity, whereas NRPs, assembled by multi-enzyme complexes, allow for diverse chemical modifications that expand functional diversity. Advances in genomics, metabolomics, high-throughput screening, and ML now enable the discovery of novel AMPs from previously unexplored or synthetic sources, including extinct organisms and *in silico* peptide libraries. These high-throughput and computational approaches are accelerating the identification of AMPs with specific structural and functional properties, ultimately facilitating the rational design of peptides capable of overcoming conventional antibiotic limitations.

Structurally, AMPs are characterized by cationicity, amphipathicity, and hydrophobicity, which enable selective targeting of negatively charged microbial membranes while sparing host cells. This selective interaction underlies their broad-spectrum activity against bacteria, fungi, viruses, and even cancer cells, while simultaneously minimizing the likelihood of resistance development. The structural diversity of AMPs encompassing α-helical, β-sheet, αβ, cyclic, and non-αβ conformations provides flexibility for dynamic membrane interactions, pore formation, and intracellular targeting, often enhanced by conserved motifs, multimerization, and PTMs. Harnessing this structural versatility through rational, structure-guided design enables the development of AMPs with enhanced potency, selectivity, and stability, offering a valuable arsenal against MDR pathogens.

SAR studies show that antimicrobial potency depends on hydrophobicity, charge, and peptide conformation. Tadpole-like AMPs and acyl-lysine oligomers rely on amphipathic structures and optimal tail/chain lengths for membrane disruption. Clovibactin and DICAMs require specific residues and backbone integrity for Gram-positive selectivity. Overall, fine-tuning hydrophobic anchoring, stereochemistry, and topology is key to effective and selective antimicrobial design.

Beyond antibacterial activity, AMPs have demonstrated significant potential as antiviral, antifungal, and antiparasitic agents. AVPs can directly target viral particles, inhibit replication, and modulate host immune responses, offering a strategy to combat emerging and drug-resistant viral infections. Similarly, AFPs selectively disrupt fungal cell walls and membranes, effectively targeting planktonic cells and resistant biofilms, and addressing the limited arsenal of existing antifungal drugs. Antiparasitic peptides exhibit potent activity against *Plasmodium*, *Leishmania*, and *Trypanosoma* species through mechanisms such as membrane disruption and enzyme inhibition, while maintaining low toxicity to host cells.

Collectively, these activities position AMPs as multifunctional therapeutics capable of addressing a wide range of infectious diseases that increasingly evade conventional treatment.

In addition to their direct antimicrobial effects, AMPs play a crucial role in modulating the host immune system. They recruit and activate immune cells, regulate cytokine production, and activate complement pathways, thereby enhancing microbial clearance while controlling excessive inflammation. Synthetic derivatives, such as IDRs, can further suppress harmful inflammatory responses in chronic infections and autoimmune conditions. The integration of antimicrobial and immunomodulatory properties provides an opportunity to develop next-generation therapeutics that not only target pathogens directly but also enhance the host’s intrinsic defense mechanisms. AMPs also influence gut microbiota composition, contributing to immune homeostasis and pathogen defense, yet this critical aspect remains underexplored. There is a research gap to address whether broad-spectrum AMPs pose risks similar to traditional antibiotics, such as microbiome disruption and dysbiosis, especially when administered systemically or orally.

Despite their promise, bacterial resistance to AMPs remains a significant concern. Microbes employ sophisticated mechanisms to counter AMP activity, including proteolytic degradation, membrane and cell wall modifications, efflux pump-mediated expulsion, and biofilm formation. Regulatory systems such as PhoP/PhoQ and sigma factors enhance bacterial adaptability under AMP-induced stress, while extracellular slime matrices and capsule production limit peptide access to target sites. Although the emergence of high-level AMP resistance is generally slower compared to conventional antibiotics, careful design, strategic administration, and combination therapies are essential to maintain therapeutic efficacy. Utmost care must be taken that the AMPs donot become like the antibiotics that developed resistant against the pathogens at very fast rate. Therefore, understanding these resistance mechanisms is critical for the rational development of AMP-based therapeutics and for devising strategies to prolong their clinical utility.

The integration of chemotype-to-genotype strategies has revolutionized AMP discovery by linking chemical structures of microbial SMs to their corresponding BGCs. Techniques such as MS-guided peptidogenomics, genome mining, transcriptomics, and *in silico* analyses enable the identification of novel AMPs and their functional characterization. These approaches also uncover regulatory, transport, and immunity-related elements within BGCs, providing critical insights into biosynthesis, stability, and activity. Chemotype-to-genotype methodologies thus accelerate the discovery of stable, specific, and effective AMPs capable of addressing emerging MDR pathogens, bridging the gap between natural diversity and therapeutic application.

The clinical translation of AMPs has been exemplified by peptide therapeutics such as insulin, nisin, gramicidin, daptomycin, and colistin, demonstrating the broad applicability of peptides across infectious, metabolic, and oncological contexts. AMPs have shown promising antiviral and anticancer activity, selectively targeting pathogen and cancer cell membranes while sparing host tissues. Clinical trials of AMPs, including nisin, Hlf1-11, C16G2, and Omiganan, demonstrate their versatility in infection prevention, wound healing, and microbiome-targeted interventions. Despite challenges such as enzymatic degradation, short half-life, and high production costs, novel delivery methods, synthetic derivatives, and combination therapies are enhancing the efficacy, safety, and translational potential of AMPs. These developments underscore the transformative potential of AMP-based therapeutics in addressing AMR and expanding clinical treatment options.

Intrinsic limitations of AMPs including susceptibility to proteolytic degradation, cytotoxicity, poor bioavailability, and pharmacokinetic challenges have constrained their widespread clinical adoption. Bacterial adaptations such as altered membrane composition, protease secretion, and biofilm formation further reduce AMP efficacy and may contribute to resistance development. High production costs and labor-intensive purification processes limit large-scale manufacturing, while pharmacological drawbacks, including antigenicity and rapid clearance, pose additional challenges. Consequently, further research is required to overcome these barriers, optimize peptide stability, and enhance clinical applicability, ensuring AMPs can achieve their full therapeutic potential.

Production and cost barriers remain central to AMP development. Traditional extraction methods yield low quantities of peptides and require extensive purification, making them expensive and impractical for large-scale use. Chemical synthesis, particularly SPPS, recombinant expression in prokaryotic and eukaryotic systems, and plant-based bioreactors offer promising solutions for scalable, cost-effective production with PTMs. Prokaryotic systems, such as *E coli*, enable high-yield, low-cost expression, while eukaryotic systems, including yeast and plant platforms, facilitate proper folding, disulfide bond formation, and glycosylation. Plant-based bioreactors, such as *N. benthamiana*, provide a low-cost and sustainable alternative for AMP production with reduced downstream processing requirements. Optimization of these production platforms is essential to meet growing clinical and commercial demand for AMP therapeutics. While scientific innovation in AMP discovery is accelerating, unclear regulatory pathways, weak patent protection, and limited economic incentives still obstruct translation into approved therapies. Addressing these structural barriers through streamlined regulation, stronger intellectual property frameworks, and sustainable reimbursement models is essential for AMPs to reach patients and fulfill their clinical potential.

Rediscovering AMPs through advanced strategies is transforming their therapeutic potential. Chemical modifications, cyclization, incorporation of unnatural amino acids, and charge engineering improve stability, protease resistance, and antimicrobial activity. Nanotechnology-based delivery systems, including nanotubes, metal nanoparticles, and lipid-based carriers, enhance bioavailability, prolong circulation, improve tissue targeting, and enable biofilm penetration. ML, AI-guided design, and computational mining accelerate discovery, optimize structure-activity relationships, and identify candidates with potent activity against multidrug-resistant pathogens. The AMP heavily relies its activity on structure, therefore, SARs studies has potential to explore the chemical structure of a AMP to serve as template and design the biological activities. Display technologies and molecular dynamics simulations further guide the rational development of AMPs with enhanced efficacy and safety. Collectively, these multidisciplinary approaches provide a robust platform for the design, optimization, and clinical translation of next-generation AMPs to combat infectious diseases, AMR, and other emerging health threats. Not only the laboratory and clinical research, but the socio-economic factors, awareness, and strong legislation of antibiotics uses are crucially important to minimized AMR in this era.

In conclusion, AMPs represent a versatile, multifunctional, and transformative class of therapeutics with immense potential to address the global challenge of AMR. Their broad-spectrum activity, structural diversity, immunomodulatory properties, and multi-target mechanisms distinguish them from conventional antibiotics, providing innovative solutions for infectious, viral, fungal, parasitic, and even oncological diseases. Despite inherent limitations, including susceptibility to degradation, pharmacokinetic challenges, and production costs, advances in chemical synthesis, recombinant expression, nanotechnology, and AI-guided discovery are rapidly overcoming these barriers. Integrating genomics, metabolomics, structure-guided design, and advanced delivery strategies promises the development of clinically translatable, effective, and safe AMPs, offering a new paradigm in therapeutic innovation and a vital weapon in the fight against MDR pathogens and emerging infectious threats. Through continued research technological innovation, and clinical translation, AMPs have the potential to redefine antimicrobial therapy and provide sustainable, next-generation solutions to the global AMR crisis. Since resistance is an ancient evolutionary process, strong policies, public awareness, proper antibiotic use, and synergistic strategies with old compounds are essential to limit its development.

## References

[B1] Abi Khattar Z. Rejasse A. Destoumieux-Garzón D. Escoubas J. M. Sanchis V. Lereclus D. . (2009). The dlt operon of *Bacillus cereus* is required for resistance to cationic antimicrobial peptides and for virulence in insects. J. Bacteriol 191, 7063–7073. doi: 10.1128/jb.00892-09, PMID: 19767427 PMC2772482

[B2] Afacan N. J. Yeung A. T. Y. Pen O. M. Hancock R. E. W. (2012). Therapeutic potential of host defense peptides in antibiotic-resistant infections. Curr. Pharm. Des. 18, 807–819. doi: 10.2174/138161212799277617, PMID: 22236127

[B3] Ahmed A. Siman-Tov G. Hall G. Bhalla N. Narayanan A. (2019). Human antimicrobial peptides as therapeutics for viral infections. Vir 11, 1–26. doi: 10.3390/v11080704, PMID: 31374901 PMC6722670

[B4] Alshrari A. S. Hudu S. A. Elmigdadi F. Imran M. (2023). The urgent threat of *Clostridioides difficile* infection: A glimpse of the drugs of the future, with related patents and prospects. Biomed 11, 426. doi: 10.3390/biomedicines11020426, PMID: 36830964 PMC9953237

[B5] Andersson D. I. Hughes D. Kubicek-Sutherland J. Z. (2016). Mechanisms and consequences of bacterial resistance to antimicrobial peptides. Drug Resist. Update 26, 43–57. doi: 10.1016/j.drup.2016.04.002, PMID: 27180309

[B6] Antimicrobial Resistance Collaborators (2022). Global burden of bacterial antimicrobial resistance in 2019: a systematic analysis. Lanc 399, 629–655. doi: 10.1016/S0140-6736(21)02724-0, PMID: 37500506

[B7] Arnison P. G. Bibb M. J. Bierbaum G. Bowers A. A. Bugni T. S. Bulaj G. . (2013). Ribosomally synthesized and post-translationally modified peptide natural products: overview and recommendations for a universal nomenclature. Nat. Prod Rep. 30, 108–160. doi: 10.1039/C2NP20085F, PMID: 23165928 PMC3954855

[B8] Ashaolu T. J. Nawaz A. Walayat N. Khalifa I. (2021). Potential “biopeptidal” therapeutics for severe respiratory syndrome coronaviruses: a review of antiviral peptides, viral mechanisms, and prospective needs. Appl. Microbiol. Biotechnol. 105, 3457–3470. doi: 10.1007/s00253-021-11267-1, PMID: 33876282 PMC8054851

[B9] Asif F. Zaman S. U. Arnab M. K. H. Hasan M. Islam M. M. (2024). Antimicrobial peptides as therapeutics: Confronting delivery challenges to optimize efficacy. Microb. 100051, 1–11. doi: 10.1016/j.microb.2024.100051

[B10] Aubry C. Goulard C. Nahori M. A. Cayet N. Decalf J. Sachse M. . (2011). OatA, a peptidoglycan O-acetyltransferase involved in *Listeria monocytogenes* immune escape, is critical for virulence. J. Infect. Dis. 204, 731–740. doi: 10.1093/infdis/jir396, PMID: 21844299 PMC3156107

[B11] Azman M. Sabri A. H. Anjani Q. K. Mustaffa M. F. Hamid K. A. (2022). Intestinal absorption study: Challenges and absorption enhancement strategies in improving oral drug delivery. Pharm. (Basel) 15, 975. doi: 10.3390/ph15080975, PMID: 36015123 PMC9412385

[B12] Bader M. W. Sanowar S. Daley M. E. Schneider A. R. Cho U. Xu W. . (2005). Recognition of antimicrobial peptides by a bacterial sensor kinase. Cell 122, 461–472. doi: 10.1016/j.cell.2005.05.030, PMID: 16096064

[B13] Bahrami A. Delshadi R. Jafari S. M. (2020). Active delivery of antimicrobial nanoparticles into microbial cells through surface functionalization strategies. Trends Food Sci. Technol. 99, 217–228. doi: 10.1016/j.tifs.2020.03.008

[B14] Band V. I. Weiss D. S. (2015). Mechanisms of antimicrobial peptide resistance in gram-negative bacteria. Antibiot 4, 18–41. doi: 10.3390/antibiotics4010018, PMID: 25927010 PMC4410734

[B15] Barra D. Simmaco M. (1995). Amphibian skin: a promising resource for antimicrobial peptides. TIBTECH 13, 205–209. doi: 10.1016/S0167-7799(00)88947-7, PMID: 7598843

[B16] Barra D. Simmaco M. Boman H. G. (1998). Gene-encoded peptide antibiotics and innate immunity. Do `animalcules’ have defense budgets? FEBS Lett. 430, 130–134. doi: 10.1016/S0014-5793(98)00494-3, PMID: 9678608

[B17] Bechinger B. (1999). The structure, dynamics, and orientation of antimicrobial peptides in membranes by multidimensional solid-state NMR spectroscopy. Biochim. Biophys. Acta 1462, 157–183. doi: 10.1016/S0005-2736(99)00205-9, PMID: 10590307

[B18] Bechinger B. Gorr S. U. (2017). Antimicrobial peptides: mechanisms of action and resistance. J. Dent. Res. 96, 254–260. doi: 10.1177/0022034516679973, PMID: 27872334 PMC5298395

[B19] Berendonk T. U. Manaia C. M. Merlin C. Fatta-Kassinos D. Cytryn E. Walsh F. . (2015). Tackling antibiotic resistance: the environmental framework. Nat. Rev. Microbiol. 13, 310–317. doi: 10.1038/nrmicro3439, PMID: 25817583

[B20] Bergholz T. M. Tang S. Wiedmann M. Boor K. J. (2013). Nisin Resistance of *Listeria monocytogenes* is increased by exposure to salt stress and is mediated via LiaR. Appl. Environ. Microbiol. 79, 5682–5688. doi: 10.1128/AEM.01797-13, PMID: 23851083 PMC3754191

[B21] Blair J. M. Webber M. A. Baylay A. J. Ogbolu D. O. Piddock L. J. . (2014). Molecular mechanisms of antibiotics resistance. Nat. Rev. Microbiol. 13, 42–51. doi: 10.1128/mBio.02825-20, PMID: 25435309

[B22] Blin K. Shaw S. Vader L. Szenei J. Reitz Z. L. Augustijn H. E. . (2025). AntiSMASH 8.0: extended gene cluster detection capabilities and analyses of chemistry, enzymology, and regulation. Nucl. Acids Res. 53, W32–W38. doi: 10.1093/nar/gkaf334, PMID: 40276974 PMC12230676

[B23] Boman H. G. (2003). Antibacterial peptides: basic facts and emerging concepts. J. Inter Med. 254, 197–215. doi: 10.1046/j.1365-2796.2003.01228.x, PMID: 12930229

[B24] Brogden K. A. (2005). Antimicrobial peptides: Pore formers or metabolic inhibitors in bacteria? Nat. Rev. Microbiol. 3, 238–249. doi: 10.1038/nrmicro1098, PMID: 15703760

[B25] Brown E. D. Wright G. D. (2016). Antibacterial drug discovery in the resistance era. Nat 529, 336–343. doi: 10.1038/nature17042, PMID: 26791724

[B26] Brunicardi J. E. H. Griffin J. H. Ferracane M. J. Kreutzer A. G. Small J. Mendoza A. T. . (2024). Structure-activity relationship studies of the peptide antibiotic clovibactin. J. Org Chem. 89, 12479–12484. doi: 10.1021/acs.joc.4c01414, PMID: 39178334 PMC11382152

[B27] Buda De Cesare G. Cristy S. A. Garsin D. A. Lorenz M. C. (2020). Antimicrobial peptides: a new frontier in antifungal therapy. mBio 11, e02123–e02120. doi: 10.1128/mBio.02123-20, PMID: 33144376 PMC7642678

[B28] Bulet P. Hetru C. Dimarcq J. L. Hoffmann D. (1999). Antimicrobial peptides in insects; structure and function. Dev. Comp. Immunol. 23, 329–344. doi: 10.1016/S0145-305X(99)00015-4, PMID: 10426426

[B29] Cardoso P. Glossop H. Meikle T. G. Aburto-Medina A. Conn C. E. Sarojini V. . (2021). Molecular engineering of antimicrobial peptides: Microbial targets, peptide motifs and translation opportunities. Biophys. Rev. 13, 35–69. doi: 10.1007/s12551-021-00784-y, PMID: 33495702 PMC7817352

[B30] Carnero Canales C. S. Marquez Cazorla J. I. Marquez Cazorla R. M. Sábio R. M. Santos H. A. Pavan F. R. . (2025). Combating Gram-negative infections: The role of antimicrobial peptides and nanotechnology in overcoming antibiotic resistance. Mater Today Bio 35, 102381. doi: 10.1016/j.mtbio.2025.102381, PMID: 41142413 PMC12546895

[B31] Carratalá J. V. Serna N. Villaverde A. Vázquez E. Ferrer-Miralles N. (2020). Nanostructured antimicrobial peptides: The last push towards clinics. Biotechnol. Adv. 44, 107603. doi: 10.1016/j.biotechadv.2020.107603, PMID: 32738381

[B32] Cereghino J. L. Cregg J. M. (2000). Heterologous protein expression in the methylotrophic yeast. Pichia pastoris. FEMS Microbiol. Rev. 24, 45–66. doi: 10.1111/j.1574-6976.2000.tb00532.x, PMID: 10640598

[B33] Cesaro A. Hoffman S. C. Das P. de la Fuente-Nunez C. (2025). Challenges and applications of artificial intelligence in infectious diseases and antimicrobial resistance. NPJ Antimicrob. Resist. 3, 2. doi: 10.1038/s44259-024-00068-x, PMID: 39843587 PMC11721440

[B34] Cesaro A. Lin S. Pardi N. de la Fuente-Nunez C. (2023). Advanced delivery systems for peptide antibiotics. Adv Drug Deliv Rev. 196, 114733. doi: 10.1016/j.addr.2023.114733, PMID: 36804008 PMC10771258

[B35] Challis G. L. (2008). Genome mining for novel natural product discovery. J. Med. Chem. 51, 2618–2628. doi: 10.1021/jm700948z, PMID: 18393407

[B36] Chaudhary S. Mahfouz M. M. (2024). Molecular farming of antimicrobial peptides. Nat. Rev. Bioeng 2, 3–5. doi: 10.1038/s44222-023-00149-y

[B37] Chen C. H. Lu T. K. (2020). Development and challenges of antimicrobial peptides for therapeutic applications. Antibiot 9 , 24. doi: 10.3390/antibiotics9010024, PMID: 31941022 PMC7168295

[B38] Chen Y. Mant C. T. Farmer S. W. Hancock R. E. Vasil M. L. Hodges R. S. (2005). Rational design of α-Helical antimicrobial peptides with enhanced activities and specificity/therapeutic index. J. Biol. Chem. 280, 12316–12329. doi: 10.1074/jbc.M413406200, PMID: 15677462 PMC1393284

[B39] Chen J. van Heel A. J. Kuipers O. P. (2020). Subcellular localization and assembly process of the nisin biosynthesis machinery in. Lactococcus lactis. mBio 11, e02825–e02820. 33173006 10.1128/mBio.02825-20 PMC7667030

[B40] Chen Y. T. Yuan Q. Shan L. T. Lin M. A. Cheng D. Q. Li C. Y. . (2013). Antitumor activity of bacterial exopolysaccharides from the endophyte *Bacillus amyloliquefaciens* sp. isolated Ophiopogon japonicus. Oncol. Lett. 5, 1787–1792. doi: 10.3892/ol.2013.1284, PMID: 23833642 PMC3700801

[B41] Chikindas M. L. Weeks R. Drider D. Chistyakov V. A. Dicks L. M. (2018). Functions and emerging applications of bacteriocins. Curr. Opin. Biotechnol. 49, 23–28. doi: 10.1016/j.copbio.2017.07.011, PMID: 28787641 PMC5799035

[B42] Chowdhury S. M. Talukder S. A. Khan A. M. Afrin N. Ali M. A. Islam R. . (2020). Antiviral peptides as promising therapeutics against SARS-CoV-2. J. Phys. Chem. B 124, 9785–9792. doi: 10.1021/acs.jpcb.0c05621, PMID: 33095007

[B43] Chu J. Vila-Farres X. Inoyama D. Ternei M. Cohen L. J. Gordon E. A. . (2016). Discovery of MRSA RSA active antibiotics using the primary sequence from the human microbiome. Nat. Chem. Biol. 12, 1004–1006. doi: 10.1038/nchembio.2207, PMID: 27748750 PMC5117632

[B44] Cimermancic P. Medema M. H. Claesen J. Kurita K. Wieland Brown L. C. Mavrommatis K. . (2014). Insights into secondary metabolism from a global analysis of prokaryotic biosynthetic gene clusters. Cell 158, 412–421. doi: 10.1016/j.cell.2014.06.034, PMID: 25036635 PMC4123684

[B45] Ciulla M. G. Gelain F. (2023). Structure-activity relationships of antibacterial peptides. Microb. Biotechnol. 16, 757–777. doi: 10.1111/1751-7915.14213, PMID: 36705032 PMC10034643

[B46] Collins B. Curtis N. Cotter P. D. Hill C. Ross R. P. (2010). The ABC transporter AnrAB contributes to the innate resistance of *Listeria monocytogenes* to nisin, bacitracin, and various beta-lactam antibiotics. Antimicrob. Agents Chemother. 54, 4416–4423. doi: 10.1128/AAC.00503-10, PMID: 20643901 PMC2944581

[B47] Cresti L. Cappello G. Pini A. (2024). Antimicrobial peptides towards clinical application- A long history to be concluded. Int. J. Mol. Sci. 25, 4870. doi: 10.3390/ijms25094870, PMID: 38732089 PMC11084544

[B48] da Cunha N. B. Cobacho N. B. Viana J. F. C. Lima L. A. Sampaio K. B. O. Dohms S. S. M. . (2017). The next generation of antimicrobial peptides (AMPs) as molecular therapeutic tools for the treatment of diseases with social and economic impacts. Drug Dis. Today 22, 234–248. doi: 10.1016/j.drudis.2016.10.017, PMID: 27890668 PMC7185764

[B49] Dando T. M. Perry C. M. (2003). Enfuvirtide. Drugs 63, 2755–2766. doi: 10.2165/00003495-200363240-00005, PMID: 14664654

[B50] Da Silva J. Leal E. C. Carvalho E. (2021). Bioactive antimicrobial peptides as therapeutic agents for infected diabetic foot ulcers. Biomol 11, 1894. doi: 10.3390/biom11121894, PMID: 34944538 PMC8699205

[B51] Davies J. (2011). How to discover new antibiotics: harvesting the parvome. Curr. Opin. Chem. Biol. 15, 5–10. doi: 10.1016/j.cbpa.2010.11.001, PMID: 21111668

[B52] D'Costa V. M. King C. E. Kalan L. Morar M. Sung W. W. Schwarz C. . (2011). Antibiotic resistance is ancient. Nat 477, 457–461. doi: 10.1038/nature10388, PMID: 21881561

[B53] de la Fuente-Nunez C. Cesaro A. Hancock R. E. W. (2023). Antibiotic failure: Beyond antimicrobial resistance. Drug Resist. Update 71, 101012. doi: 10.1016/j.drup.2023.101012, PMID: 37924726 PMC12224857

[B54] Del Olmo M. L. Andreu C. (2025). Current status of the application of antimicrobial peptides and their conjugated derivatives. Mol 30, 3070. doi: 10.3390/molecules30153070, PMID: 40807245 PMC12348590

[B55] De Lucca A. J. Walsh T. J. (1999). Antifungal peptides: novel therapeutic compounds against emerging pathogens. Antimicrob. Agents Chemother. 43, 1–11. doi: 10.1128/AAC.43.1.1, PMID: 9869556 PMC89011

[B56] Dennison S. R. Harris F. Mura M. Phoenix D. A. (2018). An atlas of anionic antimicrobial peptides from amphibians. Curr. Protein Pept. Sci. 19, 823–838. doi: 10.2174/1389203719666180226155035, PMID: 29484989

[B57] De Smet K. Contreras R. (2005). Human antimicrobial peptides: Defensins, cathelicidins, and histatins. Biotech. Lett. 27, 1337–1347. doi: 10.1007/s10529-005-0936-5, PMID: 16215847

[B58] Devrani S. Tietze D. Tietze A. A. (2025). Automated microfluidic platform for high-tthroughput biosensor development. Adv. Sensor Res. 4, 2400116. doi: 10.1002/adsr.202400116

[B59] Diamond G. Zasloff M. Eck H. Brasseur M. Maloy W. L. Bevins C. L. (1991). Tracheal antimicrobial peptide, a novel cysteine-rich peptide from mammalian tracheal mucosa: peptide isolation and cloning of a cDNA. Proc. Natl. Acad. Sci. U.S.A. 88, 3952–3956. doi: 10.1073/pnas.88.9.3952, PMID: 2023943 PMC51571

[B60] Diao L. Meibohm B. (2013). Pharmacokinetics and pharmacodynamic correlations of therapeutic peptides. Clin. Pharmacokine 52, 855–868. doi: 10.1007/s40262-013-0079-0, PMID: 23719681

[B61] Dimarcq J. L. Bulet P. Hetru C. Hoffmann J. (1998). Cysteine-rich antimicrobial peptides in invertebrates. Biopoly 47, 465–477. doi: 10.1002/(SICI)1097-0282(1998)47:6<465::AID-BIP5>3.0.CO;2-# 10333738

[B62] Divyashree M. Mani M. K. Reddy D. Kumavath R. Ghosh P. Azevedo V. . (2020). Clinical applications of antimicrobial peptides (AMPs): Where do we Stand Now? Prot Pept. Lett. 27, 120–134. doi: 10.2174/0929866526666190925152957, PMID: 31553285

[B63] Domingo-Fernández D. Gadiya Y. Preto A. J. Krettler C. A. Mubeen S. Allen A. . (2024). Natural products have increased rates of clinical trial success throughout the drug development process. J. Nat. Prod 87, 1844–1851. doi: 10.1021/acs.jnatprod.4c00581, PMID: 38970498 PMC11287737

[B64] Elad S. Epstein J. B. Raber-Durlacher J. Donnelly P. Strahilevitz J. (2012). The antimicrobial effect of iseganan HCl oral solution in patients receiving stomatotoxic chemotherapy: analysis from a multicenter, double-blind, placebo-controlled, randomized, phase III clinical trial. J. Oral. Pathol. Med. 41, 229–234. doi: 10.1111/j.1600-0714.2011.01094.x, PMID: 22077420

[B65] Elsayed Y. Y. Kühl T. Imhof D. (2025). Regulatory guidelines for the analysis of therapeutic peptides and proteins. J. Pept. Sci. 31, e70001. doi: 10.1002/psc.70001, PMID: 39921384 PMC11806371

[B66] Epand R. M. Vogel H. J. (1999). Diversity of antimicrobial peptides and their mechanisms of action. Biochim Biophys Acta. 1462(1-2):11–28. doi: 10.1016/s0005-2736(99)00198-4, PMID: 10590300

[B67] Essig A. Hofmann D. Münch D. Gayathri S. Künzler M. Kallio P. T. . (2014). Copsin, a novel peptide-based fungal antibiotic interfering with the peptidoglycan synthesis. J. Biol. Chem. 289, 34953–34964. doi: 10.1074/jbc.M114.599878, PMID: 25342741 PMC4263892

[B68] European Centre for Disease Prevention and Control (2015). “ Antimicrobial resistance surveillance in Europe 2014,” in Annual report of the European antimicrobial resistance surveillance network (EARS-Net) ( ECDC, Stockholm).

[B69] Fang P. Yu S. Ma X. Hou L. Li T. Gao K. . (2024). Applications of tandem mass spectrometry (MS/MS) in antimicrobial peptides field: Current state and new applications. Heliyon 10, e28484. doi: 10.1016/j.heliyon.2024.e28484, PMID: 38601527 PMC11004759

[B70] Fani M. Maecke H. R. Okarvi S. M. (2012). Radiolabeled peptides: valuable tools for the detection and treatment of cancer. Theranost 2, 481–501. doi: 10.7150/thno.4024, PMID: 22737187 PMC3364555

[B71] Felício M. R. Silva O. N. Gonçalves S. Santos N. C. Franco O. L. (2017). Peptides with dual antimicrobial and anticancer activities. Front. Chem. 5, 5. doi: 10.3389/fchem.2017.00005, PMID: 28271058 PMC5318463

[B72] Fieck A. Hurwitz I. Kang A. S. Durvasula R. (2010). *Trypanosoma cruzi*: synergistic cytotoxicity of multiple amphipathic anti-microbial peptides to T. cruzi potential bacterial hosts. Exp. Parasitol. 125, 342–347. doi: 10.1016/j.exppara.2010.02.016, PMID: 20206169 PMC2875304

[B73] Finking R. Marahiel M. A. (2004). Biosynthesis of nonribosomal peptides. Annu. Rev. Microbiol. 58, 453–488. doi: 10.1146/annurev.micro.58.030603.123615, PMID: 15487945

[B74] Fisher M. C. Hawkins N. J. Sanglard D. Gurr S. J. (2018). World wide emergence of resistance to antifungal drugs challenges human health and food security. Sci 360, 739–742. doi: 10.1126/science.aap7999, PMID: 29773744

[B75] Fjell C. D. Hiss J. A. Hancock R. E. Schneider G. (2012). Designing antimicrobial peptides: form follows function. Nat. Rev. Drug Dis. 11, 37–51. doi: 10.1038/nrd3591, PMID: 22173434

[B76] Flannery E. L. Chatterjee A. K. Winzeler E. A. (2013). Antimalarial drug discovery - approaches and progress towards new medicines. Nat. Rev. Microbiol. 11, 849–862. doi: 10.1038/nrmicro3138, PMID: 24217412 PMC3941073

[B77] Fleming A. (1929). On the antibacterial action of cultures of a penicillium, with special reference to their use in the isolation of B. influenza. Br. J. Exp. Pathol. 10, 226–236. PMC2566493 11545337

[B78] Fosgerau K. Hoffmann T. (2015). Peptide therapeutics: current status and future directions. Drug Dis. Today 20, 122–128. doi: 10.1016/j.drudis.2014.10.003, PMID: 25450771

[B79] Fox J. L. (2013). Antimicrobial peptides stage a comeback. Nat. Biotechnol. 31, 379–382. doi: 10.1038/nbt.2572, PMID: 23657384

[B80] Frasca L. Lande R. (2012). Role of defensins and cathelicidin LL37 in auto-immune and auto-inflammatory diseases. Curr. Pharm. Biotechnol. 13, 1882–1897. doi: 10.2174/138920112802273155, PMID: 22250708

[B81] Friedrich C. L. Moyles D. Beveridge T. J. Hancock R. E. (2000). Antibacterial action of structurally diverse cationic peptides on gram-positive bacteria. Antimicrob. Agents Chemother. 44, 2086–2092. doi: 10.1128/AAC.44.8.2086-2092.2000, PMID: 10898680 PMC90018

[B82] Friedrich C. L. Rozek A. Patrzykat A. Hancock R. E. (2001). Structure and mechanism of action of an indolicidin peptide derivative with improved activity against gram-positive bacteria. J Biol Chem. 276 (26), 24015–24022. doi: 10.1074/jbc.M009691200, PMID: 11294848

[B83] Friesen M. L. Porter S. S. Stark S. C. von Wettberg E. J. Sachs J. L. Martinez-Romero E. . (2011). Microbially mediated plant functional traits. Annu. Rev. Ecol. Evol. Syst. 42, 23–46. doi: 10.1146/annurev-ecolsys-102710-145039

[B84] Fritig B. Heitz T. Legrand M. (1998). Antimicrobial proteins in induced plant defense. Curr. Opin. Immunol. 10, 16–22. doi: 10.1016/S0952-7915(98)80025-3, PMID: 9523105

[B85] Gan B. H. Gaynord J. Rowe S. M. Deingruber T. Spring D. R. (2021). The multifaceted nature of antimicrobial peptides: current synthetic chemistry approaches and future directions. Chem. Soc. Rev. 50, 7820–7880. doi: 10.1039/D0CS00729C, PMID: 34042120 PMC8689412

[B86] Ganesan A. (2008). The impact of natural products upon modern drug discovery. Curr. Opin. Chem. Biol. 12, 306–317. doi: 10.1016/j.cbpa.2008.03.016, PMID: 18423384

[B87] Ganz T. (2003). The role of antimicrobial peptides in innate immunity. Integr. Comp. Biol. 43, 300–304. doi: 10.1093/icb/43.2.300, PMID: 21680437

[B88] Gaurav A. Bakht P. Saini M. Pandey S. Pathania R. (2023). Role of bacterial efflux pumps in antibiotic resistance, virulence, and strategies to discover novel efflux pump inhibitors. Microbiol. (Reading) 169, 001333. doi: 10.1099/mic.0.001333, PMID: 37224055 PMC10268834

[B89] Gautam A. Nanda J. S. Samuel J. S. Kumari M. Priyanka P. Bedi G. . (2016). Topical delivery of protein and peptide using novel cell penetrating peptide IMT-P8. Sci. Rep. 18, 1–13. doi: 10.1038/srep26278, PMID: 27189051 PMC4870705

[B90] Global Antimicrobial Peptides Sales Market Report (2021) ( MRRPB5), PB504212097.

[B91] Gupta A. Mumtaz S. Li C. H. Hussain I. Rotello V. M. (2019). Combatting antibiotic-resistant bacteria using nanomaterials. Chem. Soc. Rev. 48, 415–427. doi: 10.1039/C7CS00748E, PMID: 30462112 PMC6340759

[B92] Guyot N. Meudal H. Trapp S. Iochmann S. Silvestre A. Jousset G. (2020). Structure, function, and evolution of Gga-AvBD11, the archetype of the structural avian-double-β-defensin family. Proc Natl Acad Sci U S A 2020 Jan 7; 117(1):337–345. doi: 10.1073/pnas.1912941117, PMID: 31871151 PMC6955361

[B93] Haas P. Hill T. G. Wells B. R. (1938). On certain simple peptides occurring in marine algae. Biochem. J. 32, 2129–2133. doi: 10.1042/bj0322129, PMID: 16746854 PMC1264305

[B94] Hallock K. J. Lee D. K. Ramamoorthy A. (2003). MSI-78, an analogue of the magainin antimicrobial peptides, disrupts lipid bilayer structure via positive curvature strain. Biophys. J. 84, 3052–3060. doi: 10.1016/S0006-3495(03)70031-9, PMID: 12719236 PMC1302867

[B95] Hancock R. E. W. Chapple D. S. (1999). Peptides antibiotics. Antimicrob. Agents Chemo 43, 1317–1323. doi: 10.1128/AAC.43.6.1317, PMID: 10348745 PMC89271

[B96] Hancock R. E. W. Diamond G. (2000). The role of cationic antimicrobial peptides in innate host defenses. Trends Microbiol. 8, 402–410. doi: 10.1016/S0966-842X(00)01823-0, PMID: 10989307

[B97] Hancock R. E. W. Lehrer R. (1998). Cationic peptides: a new source of antibiotics. Trends Biotechnol. 16, 82–88. doi: 10.1016/S0167-7799(97)01156-6, PMID: 9487736

[B98] Harvey A. L. Ebel R. E. Quinn R. J. (2015). The reemergence of natural products for drug discovery in the genomics era. Nat. Rev. Drug Dis. 14, 111–129. doi: 10.1038/nrd4510, PMID: 25614221

[B99] Hasper H. E. Kramer N. E. Smith J. L. Hillman J. D. Zachariah C. Kuipers O. P. . (2006). An alternative bactericidal mechanism of action for lantibiotic peptides that target lipid II. Sci 313, 1636. doi: 10.1126/science.1129818, PMID: 16973881

[B100] Hendriksen R. S. Bortolaia V. Tate H. Tyson G. H. Aarestrup F. M. McDermott P. F. (2019). Using genomics to track global antimicrobial resistance. Front. Pub Health 4. doi: 10.3389/fpubh.2019.00242, PMID: 31552211 PMC6737581

[B101] Hernández-Ortiz N. Sánchez-Murcia P. A. Gil-Campillo C. Domenech M. Lucena-Agell D. Hortigüela R. . (2024). Design, synthesis and structure-activity relationship (SAR) studies of an unusual class of non-cationic fatty amine-tripeptide conjugates as novel synthetic antimicrobial agents. Front. Pharmacol. 15. doi: 10.3389/fphar.2024.1428409, PMID: 39156106 PMC11329928

[B102] Hilchie A. L. Wuerth K. Hancock R. E. (2013). Immune modulation by multifaceted cationic host defense (antimicrobial) peptides. Nat Chem Biol. 9 (12), 761–768. doi: 10.1038/nchembio.1393, PMID: 24231617

[B103] Ho C. S. Wong C. T. H. Aung T. T. Lakshminarayanan R. Mehta J. S. Rauz S. . (2024). Antimicrobial resistance: a concise update. Lanc Micro 12, 100947. doi: 10.1016/j.lanmic.2024.07.010, PMID: 39305919

[B104] Höng K. Austerlitz T. Bohlmann T. Bohlmann H. (2021). The thionin family of antimicrobial peptides. PLoS One 16 (7), e0254549. doi: 10.1371/journal.pone.0254549, PMID: 34260649 PMC8279376

[B105] Holak T. A. Engström A. Kraulis P. J. Lindeberg G. Bennich H. Jones T. A. . (1988). The solution conformation of the antibacterial peptide cecropin A: a nuclear magnetic resonance and dynamical simulated annealing study. Biochem 27, 7620–7629. doi: 10.1021/bi00420a008, PMID: 3207693

[B106] Hsu K. H. Pei C. Yeh J. Y. Shih C. H. Chung Y. C. Hung L. T. . (2009). Production of bioactive human α-defensin 5 in Pichia pastoris. J. Gen. Appl. Microbiol. 55, 395–401. doi: 10.2323/jgam.55.395, PMID: 19940385

[B107] Huan Y. Kong Q. Mou H. Yi H. (2020). Antimicrobial Peptides: Classification, design, application and research progress in multiple fields. Front. Microbiol. 11, 582779. doi: 10.3389/fmicb.2020.582779, PMID: 33178164 PMC7596191

[B108] Huang H. W. (2006). Molecular mechanism of antimicrobial peptides: The origin of cooperativity. Biochim. Biophys. Acta 1758, 1292–1302. doi: 10.1016/j.bbamem.2006.02.001, PMID: 16542637

[B109] Hughes D. Andersson D. I. (2015). Evolutionary consequences of drug resistance: shared principles across diverse targets and organisms. Nat. Rev. Gent 16, 459–471. doi: 10.1038/nrg3922, PMID: 26149714

[B110] Indira S. D. Ng N. Mukherjee P. K. (2022) A Process for the preparation of an antimicrobial peptide, PCT/IB2021/061356, 12-06-2021, 04-28-2022,WO 2022/084978 A1. USA: World Intellectual Property Organization.

[B111] Jansson J. K. Tas N. (2014). The microbial ecology of permafrost. Nat. Rev. Microbiol. 12, 414–245. doi: 10.1038/nrmicro3262, PMID: 24814065

[B112] Jenssen H. Hamill P. Hancock R. E. W. (2006). Peptide antimicrobial agents. Clin. Microbiol. Rev. 19, 491–511. doi: 10.1128/CMR.00056-05, PMID: 16847082 PMC1539102

[B113] Jones K. E. Patel N. G. Levy M. A. Storeygard A. Balk D. Gittleman J. L. . (2008). Global trends in emerging infectious diseases. Nat 451, 990–993. doi: 10.1038/nature06536, PMID: 18288193 PMC5960580

[B114] Josephson K. Ricardo A. Szostak J. W. (2014). mRNA display: from basic principles to macrocycle drug discovery. Drug Disco Today 19, 388–399. doi: 10.1016/j.drudis.2013.10.011, PMID: 24157402

[B115] Kaplan C. W. Sim J. H. Shah K. R. Kolesnikova-Kaplan A. Shi W. Eckert R. (2011). Selective membrane disruption: Mode of action of C16G2, a specifically targeted antimicrobial peptide. Antimicrob. Agents Chemother. 55, 3446–3452. doi: 10.1128/AAC.00342-11, PMID: 21518845 PMC3122425

[B116] Kaspar A. A. Reichert J. M. (2013). Future directions for peptide therapeutics development. Drug Discov. Today 18, 807–817. doi: 10.1016/j.drudis.2013.05.011, PMID: 23726889

[B117] Katz E. Demain A. L. (1977). The peptide antibiotics of Bacillus: chemistry, biogenesis, and possible functions. Bacteriol Rev. 41, 449–74. doi: 10.1128/br.41.2.449-474.1977, PMID: 70202 PMC414008

[B118] Kelly S. M. Price N. C. (2000). The use of circular dichroism in the investigation of protein structure and function. Curr. Prot Pept. Sci. 1, 349–384. doi: 10.2174/1389203003381315, PMID: 12369905

[B119] Kersten R. D. Yang Y. L. Xu Y. Cimermancic P. Nam S. J. Fenical W. . (2011). A mass spectrometry-guided genome mining approach for natural product peptidogenomics. Nat. Chem. Biol. 7, 794–802. doi: 10.1038/nchembio.684, PMID: 21983601 PMC3258187

[B120] Klaenhammer T. R. (1988). Bacteriocins of lactic acid bacteria. Biochim 70, 337–349. doi: 10.1016/0300-9084(88)90206-4, PMID: 3139051

[B121] Kong D. Hua X. Zhou R. Cui J. Wang T. Kong F. . (2022). Antimicrobial and anti-Inflammatory activities of MAF-1-derived antimicrobial peptide Mt6 and its D-enantiomer D-Mt6 against *acinetobacter baumannii* by targeting cell membranes and lipopolysaccharide interaction. Microbiol. Spectr. 10, e0131222. doi: 10.1128/spectrum.01312-22, PMID: 36190276 PMC9603722

[B122] Kudrimoti M. Curtis A. Azawi S. Worden F. Katz S. Adkins D. . (2016). Dusquetide: A novel innate defence regulator demonstrating a significant and consistent reduction in the duration of oral mucositis in preclinical data and a randomized, placebo-controlled phase 2a clinical study. J. Biotechnol. 239, 115–125. doi: 10.1016/j.jbiotec.2016.10.010, PMID: 27746305

[B123] Kumar P. Kizhakkedathu J. N. Straus S. K. (2018). Antimicrobial peptides: diversity, mechanism of action and strategies to improve the activity and biocompatibility. vivo. Biomol 8, 4. doi: 10.3390/biom8010004, PMID: 29351202 PMC5871973

[B124] Lam K. S. (2007). New aspects of natural products in drug discovery. Trends Microbiol. 15, 279–289. doi: 10.1016/j.tim.2007.04.001, PMID: 17433686

[B125] Larsson D. G. J. Flach C. F. (2022). Antibiotic resistance in the environment. Nat. Rev. Microbiol. 20, 257–269. doi: 10.1038/s41579-021-00649-x, PMID: 34737424 PMC8567979

[B126] Lee T. H. Hall K. N. Aguilar M. I. (2016). Antimicrobial peptide structure and mechanism of action: a focus on the role of membrane structure. Curr. Top. Med. Chem. 16, 25–39. doi: 10.2174/1568026615666150703121700, PMID: 26139112

[B127] Lee H. Lim S. I. Shin S. H. Lim Y. Koh J. W. Yang S. (2019). Conjugation of cell-penetrating peptides to antimicrobial peptides enhances antibacterial activity. ACS Omega 4, 15694–15701. doi: 10.1021/acsomega.9b02278, PMID: 31572872 PMC6761801

[B128] Lee D. G. Shin S. Y. Maeng C. Y. Jin Z. Z. Kim K. L. Hahm K. S. (1999). Isolation and characterization of a novel antifungal peptide from Aspergillus Niger. Biochem. Biophys. Res. Comm 263, 646–651. doi: 10.1006/bbrc.1999.1428, PMID: 10512732

[B129] Lee YJ Shirkey J. D. Park J. Bisht K. Cowan A. J. (2022). An overview of antiviral peptides and rational biodesign considerations. BioDesign Res. 2022, 9898241. doi: 10.34133/2022/9898241, PMID: 37850133 PMC10521750

[B130] Lee K. Willi J. A. Cho N. Kim I. Jewett M. C. Lee J. (2023). Cell-free biosynthesis of peptidomimetics. Biotechnol. Bioprocess Eng. 3, 1–17. doi: 10.1007/s12257-022-0268-5, PMID: 36778039 PMC9896473

[B131] Leite M. L. Sampaio K. B. Costa F. F. Franco O. L. Dias S. C. Cunha N. B. (2019). Molecular farming of antimicrobial peptides: available platforms and strategies for improving protein biosynthesis using modified virus vectors. Acad. Bras. Cienc 91, e20180124. doi: 10.1590/0001-3765201820180124, PMID: 30365717

[B132] Lemire J. A. Harrison J. J. Turner R. J. (2013). Antimicrobial activity of metals: Mechanisms, molecular targets, and applications. Nat. Rev. Microbiol. 11, 371–384. doi: 10.1038/nrmicro3028, PMID: 23669886

[B133] Li M. Lai Y. Villaruz A. E. Cha D. J. Sturdevant D. E. Otto M. (2007). Gram-positive three-component antimicrobial peptide-sensing system. Proc. Natl. Acad. Sci. U.S.A. 104, 9469–9474. doi: 10.1073/pnas.0702159104, PMID: 17517597 PMC1890518

[B134] Li W. F. Ma G. X. Zhou X. X. (2006). Apidaecin-type peptides: biodiversity, structure-function relationships and mode of action. Pept 27, 2350–2359. doi: 10.1016/j.peptides.2006.03.016, PMID: 16675061

[B135] Ling L. L. Schneider T. Peoples A. J. Spoering A. L. Engels I. Conlon B. P. . (2015). A new antibiotic kills pathogens without detecta ble resistance. Nat 517, 455–459. doi: 10.1038/nature14098, PMID: 25561178 PMC7414797

[B136] Liu Y. Zhu Y. Sun X. Ma T. Lao X. Zheng H. (2023). DRAVP: A comprehensive database of antiviral peptides and proteins. Vir 15, 820. doi: 10.3390/v15040820, PMID: 37112801 PMC10141206

[B137] Lopes B. S. Hanafiah A. Nachimuthu R. Muthupandian S. Md Nesran Z. N. Patil S. (2022). The role of antimicrobial peptides as antimicrobial and antibiofilm agents in tackling the silent pandemic of antimicrobial resistance. Mol 27, 2995. doi: 10.3390/molecules27092995, PMID: 35566343 PMC9105241

[B138] Lu Z. Liang X. Deng W. Liu Q. Wang Y. Liu M. . (2025). Studies on the antibacterial activity of the antimicrobial peptide Mastoparan X against methicillin-resistant. Staphylococcus aureus. Front. Cell Infect. Microbiol. 15. doi: 10.3389/fcimb.2025.1552872, PMID: 40510797 PMC12159006

[B139] Lubelski J. Rink R. Khusainov R. Moll G. N. Kuipers O. P. (2008). Biosynthesis, immunity, regulation, mode of action and engineering of the model lantibiotic nisin. Cell Mol. Life Sci. 65, 455–476. doi: 10.1007/s00018-007-7171-2, PMID: 17965835 PMC11131864

[B140] Lyu Z. Yang P. Lei J. Zhao J. (2023). Biological function of antimicrobial peptides on suppressing pathogens and improving host immunity. Antibiot 12, 1037. doi: 10.3390/antibiotics12061037, PMID: 37370356 PMC10295117

[B141] Ma Y. Guo Z. Xia B. Zhang Y. Liu X. Yu Y. . (2022). Identification of antimicrobial peptides from the human gut microbiome using deep learning. Nat. Biotechnol. 40, 921–931. doi: 10.1038/s41587-022-01226-0, PMID: 35241840

[B142] Mader J. S. Hoskin D. W. (2006). Cationic antimicrobial peptides as novel cytotoxic agents for cancer treatment. Exp. Opin. Investig. Drugs 15, 933–946. doi: 10.1517/13543784.15.8.933, PMID: 16859395

[B143] Madsen C. T. Refsgaard J. C. Teufel F. G. Kjærulff S. K. Wang Z. Meng G. . (2022). Combining mass spectrometry and machine learning to discover bioactive peptides. Nat. Commun. 13, 6235. doi: 10.1038/s41467-022-34031-z, PMID: 36266275 PMC9584923

[B144] Magana M. Pushpanathan M. Santos A. L. Leanse L. Fernandez M. Ioannidis A. . (2020). The value of antimicrobial peptides in the age of resistance. Lanc Infect. Dis. 20, e216–e230. doi: 10.1016/S1473-3099(20)30327-3, PMID: 32653070

[B145] Malanovic N. Lohner K. (2016). Antimicrobial peptides targeting gram-positive bacteria. Pharm. (Basel) 9, 59. doi: 10.3390/ph9030059, PMID: 27657092 PMC5039512

[B146] Malmsten M. (2014). Antimicrobial peptides. Ups J Med Sci 119 (2), 199-204. doi: 10.3109/03009734.2014.899278, PMID: 24758244 PMC4034559

[B147] Maria-Neto S. de Almeida K. C. Macedo M. L. Franco O. L. (2015). Understanding bacterial resistance to antimicrobial peptides: From the surface to deep inside. Biochim. Biophys. Acta 1848, 3078–3088. doi: 10.1016/j.bbamem.2015.02.017, PMID: 25724815

[B148] Marion D. Zasloff M. Bax A. (1988). A two-dimensional NMR study of the antimicrobial 98. magainin 2. FEBS 227, 21–26. doi: 10.1016/0014-5793(88)81405-4, PMID: 3338566

[B149] Maróti G. Kereszt A. Kondorosi E. Mergaert P. (2011). Natural roles of antimicrobial peptides in microbes, plants, and animals. Res. Microbiol. 162, 363–374. doi: 10.1016/j.resmic.2011.02.005, PMID: 21320593

[B150] Martin-Loeches I. Dale G. E. Torres A. (2018). Murepavadin: a new antibiotic class in the pipeline. Expert Rev. Anti Infect. Ther. 16, 259–268. doi: 10.1080/14787210.2018.1441024, PMID: 29451043

[B151] Matejuk A. Leng Q. Begum M. D. Woodle M. C. Scaria P. Chou S. T. . (2010). Peptide-based antifungal therapies against emerging infections. Drugs Futur. 35, 197. doi: 10.1358/dof.2010.35.3.1452077, PMID: 20495663 PMC2873032

[B152] Mathieu C. Gillard P. Benhalima K. (2017). Insulin analogues in type 1 diabetes mellitus: getting better all the time. Nat. Rev. Endocrinol. 13, 385–399. doi: 10.1038/nrendo.2017.39, PMID: 28429780

[B153] Matsuzaki K. Harada M. Handa T. Funakoshi S. Fujii N. Yajima H. . (1989). Magainin 1-induced leakage of entrapped calcein out of negatively-charged lipid vesicles. Biochim. Biophys. Acta 981, 130–134. doi: 10.1016/0005-2736(89)90090-4, PMID: 2719968

[B154] Mattanovich D. Branduardi P. Dato L. Gasser B. Sauer M. Porro D. (2012). Recombinant protein production in yeasts. Meth Mol. Biol. 824, 329–358. doi: 10.1007/978-1-61779-433-9_17, PMID: 22160907

[B155] McAuliffe O. Ross R. P. Hill C. (2001). Lantibiotics: structure, biosynthesis and mode of action. FEMS Microbiol Rev. 25, 285–308. doi: 10.1111/j.1574-6976.2001.tb00579.x, PMID: 11348686

[B156] McIntosh J. A. Donia M. S. Schmidt E. W. (2009). Ribosomal peptide natural products: bridging the ribosomal and nonribosomal worlds. Nat. Prod Rep. 26, 537–559. doi: 10.1039/b714132g, PMID: 19642421 PMC2975598

[B157] Meireles D. Pombinho R. Carvalho F. Sousa S. Cabanes D. (2020). *Listeria monocytogenes* wall teichoic acid glycosylation promotes surface anchoring of virulence factors, resistance to antimicrobial peptides, and decreased susceptibility to antibiotics. Path 9, 290. doi: 10.3390/pathogens9040290, PMID: 32316182 PMC7238011

[B158] Melo M. N. Dugourd D. Castanho M. A. (2006). Omiganan pentahydrochloride in the front line of clinical applications of antimicrobial peptides. Rec Pat. Antiinfect Drug Discov. 1, 201–207. doi: 10.2174/157489106777452638, PMID: 18221145

[B159] Mercer D. K. Robertson J. C. Miller L. Stewart C. S. O'Neil D. A. (2020). NP213 (Novexatin^®^): A unique therapy candidate for onychomycosis with a differentiated safety and efficacy profile. Med. Mycol 58, 1064–1072. doi: 10.1093/mmy/myaa015, PMID: 32232410 PMC7657096

[B160] Merrifield R. B. (1963). Solid phase peptide synthesis. I. The synthesis of a tetrapeptide. J. Am. Chem. Soc. 85, 2149–2154. doi: 10.1021/ja00897a025

[B161] Mihajlovic M. Lazaridis T. (2010). Antimicrobial peptides in toroidal and cylindrical pores. Biochim. Biophys. Acta 1798, 1485–1493. doi: 10.1016/j.bbamem.2010.04.004, PMID: 20403332 PMC2885466

[B162] Mohammad H. Thangamani S. Seleem M. N. (2015). Antimicrobial peptides and peptidomimetics - potent therapeutic allies for *Staphylococcal* infections. Curr. Pharm. Des. 21, 2073–2088. doi: 10.2174/1381612821666150310102702, PMID: 25760338 PMC8686049

[B163] Mohr H. Kleinkauf H. (1978). Alamethicin biosynthesis acetylation of the amino terminus and attachment of phenylalaninol. Biochim. Biophys. Acta 526, 375–386. doi: 10.1016/0005-2744(78)90129-8, PMID: 568941

[B164] Mookherjee N. Anderson M. A. Haagsman H. P. Davidson D. J. (2020). Antimicrobial host defence peptides: functions and clinical potential. Nat. Rev. Drug Discov. 19, 311–332. doi: 10.1038/s41573-019-0058-8, PMID: 32107480

[B165] Mora C. McKenzie T. Gaw I. M. Dean J. M. von Hammerstein H. Knudson T. A. . (2022). Over half of known human pathogenic diseases can be aggravated by climate change. Nat. Clim Chang 12, 869–875. doi: 10.1038/s41558-022-01426-1, PMID: 35968032 PMC9362357

[B166] Morar M. Wright G. D. (2010). The genomic enzymology of antibiotic resistance. Annu. Rev. Genet. 44, 25–51. doi: 10.1146/annurev-genet-102209-163517, PMID: 20822442

[B167] Morgan D. J. Okeke I. N. Laxminarayan R. Perencevich E. N. Weisenberg S. (2011). Non-prescription antimicrobial use worldwide: A systematic review. Lanc Infect. Dis. 11, 692–701. doi: 10.1016/S1473-3099(11)70054-8, PMID: 21659004 PMC3543997

[B168] Morimoto K. Yamaguchi H. Iwakura Y. Miyazaki M. Nakatani E. Iwamoto T. . (1991). Effects of proteolytic enzyme inhibitors on the nasal absorption of vasopressin and an analogue. Pharm Res 8 (9), 1175–1179. doi: 10.1023/a:1015862603939, PMID: 1724082

[B169] Muncaster S. Kraakman K. Gibbons O. Mensink K. Forlenza M. Jacobson G. . (2018). Antimicrobial peptides within the Yellowtail Kingfish (*Seriola lalandi*). Dev. Comp. Immunol. 80, 67–80. doi: 10.1016/j.dci.2017.04.014, PMID: 28433529

[B170] Musa M. Radman M. Krisko A. (2016). Decreasing translation error rate in *Escherichia coli* increases protein function. BMC Biotechnol. 16, 28. doi: 10.1186/s12896-016-0259-8, PMID: 26969280 PMC4788870

[B171] Muttenthaler M. King G. F. Adams D. J. Alewood P. F. (2021). Trends in peptide drug discovery. Nat. Rev. Drug Discov. 20, 309–325. doi: 10.1038/s41573-020-00135-8, PMID: 33536635

[B172] Nawrocki K. L. Crispell E. K. McBride S. M. (2014). Antimicrobial peptide resistance mechanisms of gram positive bacteria. Antibiot (Basel) 3, 461–492. doi: 10.3390/antibiotics3040461, PMID: 25419466 PMC4239024

[B173] Nawrot R. Barylski J. Nowicki G. Broniarczyk J. Buchwald W. Goździcka-Józefiak A. (2014). Plant antimicrobial peptides. Folia Microbiol. 2014, 181–196. doi: 10.1007/s12223-013-0280-4, PMID: 24092498 PMC3971460

[B174] Newman D. J. Cragg G. M. (2020). Natural Products as Sources of New Drugs over the Nearly Four Decades from 01/1981 to 09/2019. J Nat Prod 83 (3), 770-803. doi: 10.1021/acs.jnatprod.9b01285, PMID: 32162523

[B175] Ngashangva N. Mukherjee P. Sharma K. C. Kalita M. C. Indira S. (2021). Analysis of antimicrobial peptide metabolome of bacterial endophyte isolated from traditionally used medicinal plant *Millettia pachycarpa* Benth. Front. Microbiol. 12, 656896. doi: 10.3389/fmicb.2021.656896, PMID: 34149644 PMC8208310

[B176] Ngashangva N. Mukherjee P. K. Sharma C. Kalita M. C. Sarangthem I. (2022). Integrated genomics and proteomics analysis of *Paenibacillus peoriae* IBSD35 and insights into its antimicrobial characteristics. Sci. Rep. 12, 18861. doi: 10.1038/s41598-022-23613-y, PMID: 36344671 PMC9640621

[B177] Nguyen L. T. Haney E. F. Vogel H. J. (2011). The expanding scope of antimicrobial peptide structures and their modes of action. Trends Biotech. 29, 464–472. doi: 10.1016/j.tibtech.2011.05.001, PMID: 21680034

[B178] Nijman S. M. B. (2015). Functional genomics to uncover drug mechanism of action. Nat. Chem. Biol. 11, 942–948. doi: 10.1038/nchembio.1963, PMID: 26575241

[B179] Nijnik A. Madera L. Ma S. Waldbrook M. Elliott M. R. Easton D. M. . (2010). Synthetic cationic peptide IDR-1002 provides protection against bacterial infections through chemokine induction and enhanced leukocyte recruitment. J. Immunol. 184, 2539–2550. doi: 10.4049/jimmunol.0901813, PMID: 20107187

[B180] Olano J. P. Weller P. F. Guerrant R. L. Walker D. H. (2011). Principles of parasitism: host-parasite interactions. In Tropl Infect. Dis. 2011, 1–7. doi: 10.1016/B978-0-7020-3935-5.00001-X

[B181] Oliveira Júnior N. G. Souza C. M. Buccini D. F. Cardoso M. H. Franco O. L. (2025). Antimicrobial peptides: structure, functions and translational applications. Nat. Rev. Microbiol. 23, 687–700. doi: 10.1038/s41579-025-01200-y, PMID: 40646173

[B182] Oman T. J. van der Donk W. A. (2010). Follow the leader: The use of leader peptides to guide natural product biosynthesis. Nat. Chem. Biol. 6, 9–18. doi: 10.1038/nchembio.286, PMID: 20016494 PMC3799897

[B183] Ostaff M. J. Stange E. F. Wehkamp J. (2013). Antimicrobial peptides and gut microbiota in homeostasis and pathology. EMBO Mol. Med. 5, 1465–1483. doi: 10.1002/emmm.201201773, PMID: 24039130 PMC3799574

[B184] Ourth D. D. (2004). Antiviral activity against human immunodeficiency virus-1 *in vitro* by myristoylated-peptide from *Heliothis virescens*. Biochem. Biophys. Res. Commun. 320, 190–196. doi: 10.1016/j.bbrc.2004.05.137, PMID: 15207720

[B185] Pane K. Durante L. Crescenzi O. Cafaro V. Pizzo E. Varcamonti M. . (2017). Antimicrobial potency of cationic antimicrobial peptides can be predicted from their amino acid composition: Application to the detection of “Cryptic” antimicrobial peptides. J. Theor. Biol. 419, 254–265. doi: 10.1016/j.jtbi.2017.02.012, PMID: 28216428

[B186] Papagianni M. (2003). Ribosomally synthesized peptides with antimicrobial properties: biosynthesis, structure, function, and applications. Biotech. Adv. 21, 465–499. doi: 10.1016/S0734-9750(03)00077-6, PMID: 14499150

[B187] Passioura T. Katoh T. Goto Y. Suga H. (2014). Selection-based discovery of drug like macrocyclic peptides. Annu. Rev. Biochem. 83, 727–752. doi: 10.1146/annurev-biochem-060713-035456, PMID: 24580641

[B188] Pasupuleti M. Schmidtchen A. Chalupka A. Ringstad L. Malmsten M. (2009). End-Tagging of ultra-short antimicrobial peptides by W/F stretches to facilitate bacterial killing. PloS One 4, 5285. doi: 10.1371/journal.pone.0005285, PMID: 19381271 PMC2667214

[B189] Peschel A. Sahl H. G. (2006). The co-evolution of host cationic antimicrobial peptides and microbial resistance. Nat. Rev. Microbiol. 4, 529–536. doi: 10.1038/nrmicro1441, PMID: 16778838

[B190] Pillay S. Calderón-Franco D. Urhan A. Abeel T. (2022). Metagenomic-based surveillance systems for antibiotic resistance in non-clinical settings. Front. Microbiol. 13. doi: 10.3389/fmicb.2022.1066995, PMID: 36532424 PMC9755710

[B191] Pitale D. M. Kaur G. Baghel M. Kaur K. J. Shaha C. (2020). Halictine-2 antimicrobial peptide shows promising anti-parasitic activity against *Leishmania* spp. Exp. Parasitol. 218, 107987. doi: 10.1016/j.exppara.2020.107987, PMID: 32891601

[B192] Powers J. P. S. Hancock R. E. W. (2003). The relationship between peptide structure and antibacterial activity. Pept 24, 1681–1691. doi: 10.1016/j.peptides.2003.08.023, PMID: 15019199

[B193] Price G. Patel D. A. (2023). “ Drug bioavailability,” in StatPearls ( StatPearls Publishing, Treasure Island (FL). 32496732

[B194] Pu J. Wang Q. Xu W. Lu L. Jiang S. (2019). Development of protein- and peptide-based HIV entry inhibitors targeting gp120 or gp41. Vir 11, 705. doi: 10.3390/v11080705, PMID: 31374953 PMC6722851

[B195] Qiu S. Yi H. Hu J. Cao Z. Wu Y. Li W. (2012). The binding mode of fusion inhibitor T20 onto HIV-1 gp41 and relevant T20-resistant mechanisms explored by computational study. Curr. HIV Res. 10, 182–194. doi: 10.2174/157016212799937191, PMID: 22339124

[B196] Radzishevsky I. S. Kovachi T. Porat Y. Ziserman L. Zaknoon F. Danino D. . (2008). Structure-activity relationships of antibacterial acyl-lysine oligomers. Chem. Biol. 15, 354–362. doi: 10.1016/j.chembiol.2008.03.006, PMID: 18420142

[B197] Reddy K. V. R. Yedery R. D. Aranha C. (2004). Antimicrobial peptides: premises and promises. Int. J. Antimicrob. Agents 24, 536–547. doi: 10.1016/j.ijantimicag.2004.09.005, PMID: 15555874

[B198] Reimann O. Seitz O. Sarma D. Zitterbart R. (2019). A traceless catch-and-release method for rapid peptide purification. J. Pept. Sci. 25, e3136. doi: 10.1002/psc.3136, PMID: 30479039

[B199] Riley M. A. Wertz J. E. (2002). Bacteriocins: evolution, ecology, and application. Annu. Rev. Microbiol. 56, 117–137. doi: 10.1146/annurev.micro.56.012302.161024, PMID: 12142491

[B200] Roque-Borda C. A. Bento da Silva P. Rodrigues M. C. Di Filippo L. D. Duarte J. L. Chorilli M. . (2022). Pharmaceutical nanotechnology: Antimicrobial peptides as potential new drugs against WHO list of critical, high, and medium priority bacteria. Eur. J. Med. Chem. 5, 241:114640. doi: 10.1016/j.ejmech.2022.114640, PMID: 35970075

[B201] Rosenblueth M. Martinez-Romero E. (2006). Bacterial endophytes and their interactions with hosts. Mol. Plant-Microbe Inter 19, 827–837. doi: 10.1094/MPMI-19-0827, PMID: 16903349

[B202] Rozek T. Bowie J. H. Wallace J. C. Tyler M. J. (2000). The antibiotic and anticancer active aurein peptides from the Australian Bell Frogs Litoria aurea and Litoria raniformis. Part 2. Sequence determination using electrospray mass spectrometry. Rapid Commun Mass Spectrom 14(21):2002–2011. doi: 10.1002/1097-0231(20001115)14:21<2002::AID-RCM128>3.0.CO;2-3, PMID: 11085410

[B203] Sadeeq M. Li Y. Wang C. Hou F. Zuo J. Xiong P. (2025). Unlocking the power of antimicrobial peptides: advances in production, optimization, and therapeutics. Front. Cell Infect. Microbiol. 15. doi: 10.3389/fcimb.2025.1528583, PMID: 40365533 PMC12070195

[B204] Sanguinetti M. Posteraro B. Lass-Flörl C. (2015). Antifungal drug resistance among *Candida* species: mechanisms and clinical impact. Myc 58, 2–13. doi: 10.1111/myc.12330, PMID: 26033251

[B205] Santos-Júnior C. D. Torres M. D. T. Duan Y. Rodríguez Del Río Á. Schmidt T. S. B. Chong H. . (2024). Discovery of antimicrobial peptides in the global microbiome with machine learning. Cell 187, 3761–3778.e16. doi: 10.1016/j.cell.2024.05.013, PMID: 38843834 PMC11666328

[B206] Sato Y. Unno Y. Ubagai T. Ono Y. (2018). Sub-minimum inhibitory concentrations of colistin and polymyxin B promote *Acinetobacter baumannii* biofilm formation. PloS One 13, e0194556. doi: 10.1371/journal.pone.0194556, PMID: 29554105 PMC5858813

[B207] Schaberla T. F. Hack I. M. (2014). Overcoming the current deadlock in antibiotic research. Trends Microbiol. 22, 165–167. doi: 10.1016/j.tim.2013.12.007, PMID: 24698433

[B208] Schibli D. J. Nguyen L. T. Kernaghan S. D. Rekdal Ø. Vogel H. J. (2006). Structure-function analysis of tritrpticin analogs: potential relationships between antimicrobial activities, model membrane interactions, and their micelle-bound NMR structures. Biophys. J. 91, 4413–4426. doi: 10.1529/biophysj.106.085837, PMID: 16997878 PMC1779919

[B209] Schorn M. A. Verhoeven S. Ridder L. Huber F. Acharya D. D. Aksenov A. A. . (2021). A community resource for paired genomic and metabolomic data mining. Nat. Chem. Biol. 17, 363–368. doi: 10.1038/s41589-020-00724-z, PMID: 33589842 PMC7987574

[B210] Sehnal L. Brammer-Robbins E. Wormington A. M. Blaha L. Bisesi J. Larkin I. . (2021). Microbiome composition and function in aquatic vertebrates: Small organisms making big impacts on aquatic animal health. Front Microbiol 12, 567408. doi: 10.3389/fmicb.2021.567408, PMID: 33776947 PMC7995652

[B211] Seiple I. B. Zhang Z. Jakubec P. Langlois-Mercier A. Wright P. M. Hog D. T. . (2016). A platform for the discovery of new macrolide antibiotics. Nat 5, 338–345. doi: 10.1038/nature17967, PMID: 27193679 PMC6526944

[B212] Shahabuddin M. Fields I. Bulet P. Hoffmann J. A. Miller L. H. (1998). *Plasmodium gallinaceum*: differential killing of some mosquito stages of the parasite by insect defensin. Exp. Parasitol. 89, 103–112. doi: 10.1006/expr.1998.4212, PMID: 9603495

[B213] Shai Y. (2002). Mode of action of membrane active antimicrobial peptides. Biopoly (Peptide Science) 66, 236–248. doi: 10.1002/bip.10260, PMID: 12491537

[B214] Shanmugaraj B. Bulaon C. J. I. Malla A. Phoolcharoen W. (2021). Biotechnological insights on the expression and production of antimicrobial peptides in plants. Mol 26, 4032. doi: 10.3390/molecules26134032, PMID: 34279372 PMC8272150

[B215] Shriwastav S. Kaur N. Hassan M. Ahmed Mohammed S. Chauhan S. Mittal D. . (2025). Antimicrobial peptides: a promising frontier to combat antibiotic resistant pathogens. Ann. Med. Surg. (Lond); 87, 2118–2132. doi: 10.1097/MS9.0000000000003106, PMID: 40212220 PMC11981355

[B216] Siegers K. Heinzmann S. Entian K.-D. (1996). Biosynthesis of lantibiotic nisin. J. Biol. Chem. 271, 12294–12301. doi: 10.1074/jbc.271.21.12294, PMID: 8647829

[B217] Silva J. P. Appelberg R. Gama F. M. (2016). Antimicrobial peptides as novel anti-tuberculosis therapeutics. Biotechnol. Adv. 34, 924–940. doi: 10.1016/j.biotechadv.2016.05.007, PMID: 27235189

[B218] Srinivas N. Jetter P. Ueberbacher B. J. Werneburg M. Zerbe K. Steinmann J. . (2010). Peptidomimetic antibiotics target outer-membrane biogenesis in. Pseudomonas aeruginosa. Sci. 327, 1010–1013. doi: 10.1126/science.1182749, PMID: 20167788

[B219] Stein T. Heinzmann S. Düsterhus S. Borchert S. Entian K. D. (2005). Expression and functional analysis of the subtilin immunity genes spaIFEG in the subtilin-sensitive host *Bacillus subtilis* MO1099. J. Bacteriol 187, 822–828. doi: 10.1128/JB.187.3.822-828.2005, PMID: 15659659 PMC545732

[B220] Steiner H. Hultmark D. Engström A. Bennich H. Boman H. G. (1981). Sequence and specificity of two antibacterial proteins involved in insect immunity. Nat 292, 246–248. doi: 10.1038/292246a0, PMID: 7019715

[B221] Steiner L. Hirsch T. Schulte M. Kueckelhaus M. Jacobsen F. Mersch E. A. . (2012). Innate defense regulator peptide 1018 in wound healing and wound infection. PLoS One. 7 (8), e39373. doi: 10.1371/journal.pone.0039373, PMID: 22879874 PMC3412849

[B222] Stielow M. Witczyńska A. Kubryń N. Fijałkowski Ł. Nowaczyk J. Nowaczyk A. (2023). The bioavailability of drugs-The current state of knowledge. Mol 28, 8038. doi: 10.3390/molecules28248038, PMID: 38138529 PMC10745386

[B223] Stokes J. M. Yang K. Swanson K. Jin W. Cubillos-Ruiz A. Donghia N. M. . (2020). A deep learning approach to antibiotic discovery. Cell 180, 688–702.e13. doi: 10.1016/j.cell.2020.01.021, PMID: 32084340 PMC8349178

[B224] Sullivan R. Santarpia P. Lavender S. Gittins E. Liu Z. Anderson M. H. . (2011). Clinical efficacy of a specifically targeted antimicrobial peptide mouth rinse: targeted elimination of *Streptococcus mutans* and prevention of demineralization. Caries Res. 45, 415–428. doi: 10.1159/000330510, PMID: 21860239 PMC3169368

[B225] Swanson K. Liu G. Catacutan D. B. Arnold A. Zou J. Stokeset J. M. (2024). Generative AI for designing and validating easily synthesizable and structurally novel antibiotics. Nat. Mach. Intell. 6, 338–353. doi: 10.1038/s42256-024-00809-7

[B226] Szymczak P. Możejko M. Grzegorzek T. Jurczak R. Bauer M. Neubauer D. . (2023). Discovering highly potent antimicrobial peptides with deep generative model HydrAMP. Nat. Commun. 14, 1453. doi: 10.1038/s41467-023-36994-z, PMID: 36922490 PMC10017685

[B227] Tang Z. Jiang W. Li S. Huang X. Yang Y. Chen X. (2023). Design and evaluation of tadpole-like conformational antimicrobial peptides. Commun. Biol. 2023, 6:1177. doi: 10.1038/s42003-023-05560-0, PMID: 37980400 PMC10657444

[B228] Teixeira V. Feio M. J. Bastos M. (2012). Role of lipids in the interaction of antimicrobial peptides with membranes. Prog. Lipid Res. 51, 149–177. doi: 10.1016/j.plipres.2011.12.005, PMID: 22245454

[B229] Theuretzbacher U. Outterson K. Engel A. Karlén A. (2020). The global preclinical antibacterial pipeline. Nat. Rev. Microbiol. 18, 275–285. doi: 10.1038/s41579-019-0288-0, PMID: 31745331 PMC7223541

[B230] Thundimadathil J. (2012). Cancer treatment using peptides: current therapies and future prospects. J. Amino Acids 2012, 1–13. doi: 10.1155/2012/967347, PMID: 23316341 PMC3539351

[B231] Timón M. L. Andrés A. I. Otte J. Petrón M. J. (2019). Antioxidant peptides (<3 kDa) identified on hard cow milk cheese with rennet from different origin. Food Res Int 120, 643–649. doi: 10.1016/j.foodres.2018.11.019, PMID: 31000282

[B232] Tomasz A. (2006). Weapons of microbial drug resistance abound in soil flora. Sci 311, 342–343. doi: 10.1126/science.1123982, PMID: 16424327

[B233] Tong Z. Zhang Y. Ling J. Ma J. Huang L. Zhang L. (2014). An *in vitro* study on the effects of nisin on the antibacterial activities of 18 antibiotics against *Enterococcus faecalis*. PloS One 9, e89209. doi: 10.1371/journal.pone.0089209, PMID: 24586598 PMC3930635

[B234] Torcato I. M. Castanho M. A. R. B. Henriques S. T. (2012). The application of biophysical techniques to study antimicrobial peptides. Spect: Inter J. 27, 541–549. doi: 10.1155/2012/460702

[B235] Torrent M. Pulido D. Rivas L. Andreu D. (2012). Antimicrobial peptide action on parasites. Curr. Drug Targets 13, 1138–1147. doi: 10.2174/138945012802002393, PMID: 22664071

[B236] Torres M. D. T. Brooks E. F. Cesaro A. Sberro H. Gill M. O. Nicolaou C. . (2024). Mining human microbiomes reveals an untapped source of peptide antibiotics. Cell 187, 5453–5467.e15. doi: 10.1016/j.cell.2024.07.027, PMID: 39163860 PMC12821620

[B237] Torres M. D. T. Cao J. Franco O. L. Lu T. K. de la Fuente-Nunez C. (2021). Synthetic biology and computer-based frameworks for antimicrobial peptide discovery. ACS Nano 15, 2143–2164. doi: 10.1021/acsnano.0c09509, PMID: 33538585 PMC8734659

[B238] Transparency Market Research (2012). Hints: Peptide Therapeutics Market: Global Industry Analysis, Size, Share, Growth, Trends and Forecast 2012−2018. Transparency Market Research, Albany, NY.

[B239] Udwary D. W. Doering D. T. Foster B. Smirnova T. Kautsar S. A. Mouncey N. J. (2025). The secondary metabolism collaboratory: a database and web discussion portal for secondary metabolite biosynthetic gene clusters. Nucl. Acids Res. 53 (D1), D717-D723. doi: 10.1093/nar/gkae1060, PMID: 39540430 PMC11701679

[B240] Uhliga T. Kyprianoua T. Martinellia F. G. Oppici C. A. Heiligers D. Hills D. R. J. . (2014). The emergence of peptides in the pharmaceutical business: From exploration to exploitation. Eu Prot Asso Open Proteome 4, 58–69. doi: 10.1016/j.euprot.2014.05.003

[B241] van der Does A. M. Hensbergen P. J. Bogaards S. J. Cansoy M. Deelder A. M. van Leeuwen H. C. . (2012). The human lactoferrin-derived peptide hLF1–11 exerts immunomodulatory effects by specific inhibition of myeloperoxidase activity. J. Immunol. 188, 5012–5019. doi: 10.4049/jimmunol.1102777, PMID: 22523385

[B242] van der Hooft J. J. J. Mohimani H. Bauermeister A. Dorrestein P. C. Duncan K. R. Medema M. H. (2020). Linking genomics and metabolomics to chart specialized metabolic diversity. Chem. Soc. Rev. 49, 3297–3314. doi: 10.1039/d0cs00162g, PMID: 32393943

[B243] Vecchio I. Tornali C. Bragazzi N. L. Martini M. (2018). The discovery of insulin: An important milestone in the history of medicine. Front. Endocrinol. (Lausanne) 9,613. doi: 10.3389/fendo.2018.00613, PMID: 30405529 PMC6205949

[B244] Velasquez J. E. van der Donk W. A. (2011). Genome mining for ribosomally synthesized natural products. Curr. Opin. Chem. Biol. 15, 11–21. doi: 10.1016/j.cbpa.2010.10.027, PMID: 21095156 PMC3090663

[B245] Verma N. Karmakar M. Singh K. P. Smita S. (2013). Structural and dynamic insights into S100B protein activity inhibition by melittin for the treatment of epilepsy. Int. J. Comp. App NSAAILS 1, 55–60. doi: 10.1007/978-1-4939-2285-7_3, PMID: 25555720 PMC4578715

[B246] Vilas Boas L. C. P. Campos M. L. Berlanda R. L. A. de Carvalho Neves N. Franco O. L. (2019). Antiviral peptides as promising therapeutic drugs. Cell Mol Life Sci 2019 Sep; 76 (18), 3525–3542. doi: 10.1007/s00018-019-03138-w, PMID: 31101936 PMC7079787

[B247] Vilas Boas L. C. P. Campos M. L. Berlanda R. L. A. de Carvalho Neves N. Franco O. L. (2019). Antiviral peptides as promising therapeutic drugs. Cell Mol. Life Sci. 18, 3525–3542. doi: 10.1007/s00018-019-03138-w, PMID: 31101936 PMC7079787

[B248] Vizioli J. Salzet M. (2002). Antimicrobial peptides from animals: focus on invertebrates. Trends Pharmacol. Sci. 23, 494–496. doi: 10.1016/S0165-6147(02)02105-3, PMID: 12413797

[B249] Walkenhorst W. F. Klein J. W. Vo P. Wimley W. C. (2013). pH Dependence of microbe sterilization by cationic antimicrobial peptides. Antimicrob. Agents Chemother. 57, 3312–3320. doi: 10.1128/AAC.00063-13, PMID: 23650166 PMC3697317

[B250] Walsh C. T. Fischbach M. A. (2010). Natural products version 2.0: connecting genes to molecules. J. Am. Chem. Soc. 132, 2469–2493. doi: 10.1021/ja909118a, PMID: 20121095 PMC2828520

[B251] Walsh C. J. Guinane C. M. O' Toole P. W. Cotter P. D. (2017). A profile hidden Markov model to investigate the distribution and frequency of LanB-encoding lantibiotic modification genes in the human oral and gut microbiome. Peer J. 5, e3254. doi: 10.7717/peerj.3254, PMID: 28462050 PMC5410138

[B252] Wan F. Torres M. D. T. Peng J. de la Fuente-Nunez C. (2024). Deep-learning-enabled antibiotic discovery through molecular de-extinction. Nat. BioMed. Eng. 8, 854–871. doi: 10.1038/s41551-024-01201-x, PMID: 38862735 PMC11310081

[B253] Wang G. (2008). Structures of human host defense cathelicidin LL-37 and its smallest antimicrobial peptide KR-12 in lipid micelles. J. Biol. Chem. 283, 32637–32643. doi: 10.1074/jbc.M805533200, PMID: 18818205

[B254] Wang G. (2012). Post-translational modifications of natural antimicrobial peptides and strategies for peptide engineering. Curr. Biotechnol. 1, 72–79. doi: 10.2174/2211550111201010072, PMID: 24511461 PMC3914544

[B255] Wang G. (2015). Improved methods for classification, prediction, and design of antimicrobial peptides. Methods Mol. Biol. 1268, 43–66., PMID: 25555720 10.1007/978-1-4939-2285-7_3 PMC4578715

[B256] Wang C. Hong T. Cui P. Wang J. Xia J. (2021). Antimicrobial peptides towards clinical application: Delivery and formulation. Adv. Drug Delivery Rev. 175, 113818. doi: 10.1016/j.addr.2021.05.028, PMID: 34090965

[B257] Wang G. Li X. Wang Z. (2016). APD3: the antimicrobial peptide database as a tool for research and education. Nuc Acids Res. 44, D1087–D1093. doi: 10.1093/nar/gkv1278, PMID: 26602694 PMC4702905

[B258] Wang C. K. Shih L. Y. Chang K. Y. (2017). Large-scale analysis of antimicrobial activities in relation to amphipathicity and charge reveals novel characterization of antimicrobial peptides. Mol 22, 2037. doi: 10.3390/molecules22112037, PMID: 29165350 PMC6150348

[B259] Wang Y. Song M. Liu F. Liang Z. Hong R. (2025). Artificial intelligence using a latent diffusion model enables the generation of diverse and potent antimicrobial peptides. Sci. Adv. 11, 7171. doi: 10.1126/sciadv.adp7171, PMID: 39908380 PMC11797553

[B260] Wang X. Zhu M. Yang G. Su C. Zhang A. Cao R. . (2022). Therapeutic peptides: current applications and future directions. Sign Transduct Targ Ther. 7, 48. doi: 10.1038/s41392-022-00904-4, PMID: 35165272 PMC8844085

[B261] Wang G. Zietz C. M. Mudgapalli A. Wang S. Wang Z. (2011). Expression of cecropin B in. Pichia pastoris its bioactivity vitro. Exp. Therap Med. 2, 655–660. doi: 10.3892/etm.2011.262, PMID: 22977556 PMC3440743

[B262] Wang G. Zietz C. M. Mudgapalli A. Wang S. Wang Z. (2022). The evolution of the antimicrobial peptide database over 18 years: Milestones and new features. Prot Sci. 31, 92–106. doi: 10.1002/pro.4185, PMID: 34529321 PMC8740828

[B263] Wellington E. M. Boxall A. B. Cross P. Feil E. J. Gaze W. H. Hawkey P. M. . (2013). The role of the natural environment in the emergence of antibiotic resistance in Gram-negative bacteria. Lanc Infect. Dis. 13, 155–165. doi: 10.1016/S1473-3099(12)70317-1, PMID: 23347633

[B264] Wen Q. Zhang L. Zhao F. Chen Y. Su Y. Zhang X. . (2023). Production technology and functionality of bioactive peptides. Curr. Pharm. Des. 29, 652–674. doi: 10.2174/1381612829666230201121353, PMID: 36725828

[B265] White C. J. Yudin A. K. (2011). Contemporary strategies for peptide macrocyclization. Nat. Chem. 3, 509–524. doi: 10.1038/nchem.1062, PMID: 21697871

[B266] WHO (2014). Antimicrobial resistance: global report on surveillance (Geneva: World Health Organization).

[B267] WHO (2022). Bacterial vaccines in clinical and preclinical development: an overview and analysis (Geneva: World Health Organization). Licence: CC BY-NC-SA 3.0 IGO.

[B268] Wild C. Oas T. McDanal C. Bolognesi D. Matthews T. (1992). A synthetic peptide inhibitor of human immunodeficiency virus replication: correlation between solution structure and viral inhibition. Proc. Natl. Acad. Sci. U.S.A. 89, 10537–10541. doi: 10.1073/pnas.89.21.10537, PMID: 1438243 PMC50374

[B269] Wimley W. C. (2010). Describing the mechanism of antimicrobial peptide action with the interfacial activity model. ACS Chem. Biol. 5, 905–917. doi: 10.1021/cb1001558, PMID: 20698568 PMC2955829

[B270] Witek K. Nasim M. J. Bischoff M. Gaupp R. Arsenyan P. Vasiljeva J. . (2017). Selenazolinium salts as “Small Molecule Catalysts” with high potency against ESKAPE bacterial pathogens. Mol 22, 2174. doi: 10.3390/molecules22122174, PMID: 29292789 PMC6149925

[B271] Wommack A. J. Robson S. A. Wanniarachchi Y. A. Wan A. Turner C. J. Wagner G. . (2012). NMR solution structure and condition-dependent oligomerization of the antimicrobial peptide human defensin 5. Biochem 51, 9624–9637. doi: 10.1021/bi301255u, PMID: 23163963 PMC3579768

[B272] Xiang W. S. Wang J. D. Wang X. J. Bingchamides A and B. (2009). two novel cyclic pentapeptides from *Streptomyces bingchenggenesis*: Fermentation, isolation, structural elucidation and biological properties. J. Antibiot 62, 501–505. doi: 10.1038/ja.2009.60, PMID: 19609291

[B273] Xiao W. Jiang W. Chen Z. Huang Y. Mao J. Zheng W. . (2025). Advance in peptide-based drug development: delivery platforms, therapeutics and vaccines. Signal Transduct Target Ther 10 (1), 74. doi: 10.1038/s41392-024-02107-5, PMID: 40038239 PMC11880366

[B274] Yan F. Auerbach D. Chai Y. Keller L. Tu Q. Hüttel S. . (2018). Biosynthesis and heterologous production of vioprolides: rational biosynthetic engineering and unprecedented 4-methylazetidinecarboxylic acid formation. Angew Chem. Int. Ed. 57, 8754–8759. doi: 10.1002/anie.201802479, PMID: 29694699

[B275] Yang L. Harroun T. A. Weiss T. M. Ding L. Huang H. W. (2001). Barrel-stave model or toroidal model? A case study on melittin pores. Biophys. J. 81, 1475–1485. doi: 10.1016/S0006-3495(01)75802-X, PMID: 11509361 PMC1301626

[B276] Yang L. Tian Z. Zhou L. Zhu L. Sun C. Huang M. . (2022). *In vitro* antifungal activity of a novel antimicrobial peptide AMP-17 against planktonic cells and biofilms of *Cryptococcus neoformans*. Infect. Drug Resist. 15, 233–248. doi: 10.2147/IDR.S344246, PMID: 35115792 PMC8800587

[B277] Yeaman M. R. Yount N. Y. (2003). Mechanisms of antimicrobial peptide action and resistance. Pharmacol. Rev. 55, 27–55. doi: 10.1124/pr.55.1.2, PMID: 12615953

[B278] Zankari E. Hasman H. Cosentino S. Vestergaard M. Rasmussen S. Lund O. . (2012). Identification of acquired antimicrobial resistance genes. J. Antimicrob. Chemother. 67, 2640–2640. doi: 10.1093/jac/dks261, PMID: 22782487 PMC3468078

[B279] Zasloff M. (2002). Antimicrobial peptides of multicellular organisms. Nat 415, 389–395. doi: 10.1038/415389a, PMID: 11807545

[B280] Zhang Q. (2025). Antimicrobial peptides: from discovery to developmental applications. Appl. Environ. Microbiol. 91, e0211524. doi: 10.1128/aem.02115-24, PMID: 40178173 PMC12016500

[B281] Zhang H. K. Xie J. Lerner R. A. (2014). A proximity based general method for identification of ligand and receptor interactions in living cells. Biochem. Bioph Res. Co 454, 251–255. doi: 10.1016/j.bbrc.2014.10.085, PMID: 25451250

[B282] Zhang Q. Y. Yan Z. B. Meng Y. M. Hong X. Y. Shao G. Ma J. J . (2021). Antimicrobial peptides: mechanism of action, activity and clinical potential. Mil Med. Res. 8, 48. doi: 10.1186/s40779-021-00343-2, PMID: 34496967 PMC8425997

[B283] Zhang Z.-T. Zhu S.-Y. (2009). Drosomycin, an essential component of antifungal defense in drosophila. Insect Mol. Biol. 18, 549–556. doi: 10.1111/j.1365-2583.2009.00907.x, PMID: 19754735

[B284] Zheng S. Tu Y. Li B. Qu G. Li A. Peng X. . (2025). Antimicrobial peptide biological activity, delivery systems and clinical translation status and challenges. J. Transl. Med. 23, 292. doi: 10.1186/s12967-025-06321-9, PMID: 40055730 PMC11887333

[B285] Zhong C. Zhu N. Zhu Y. Liu T. Gou S. Xie J. . (2020). Antimicrobial peptides conjugated with fatty acids on the side chain of D-amino acid promises antimicrobial potency against multidrug-resistant bacteria. Eur J Pharm Sci 141:105123. doi: 10.1016/j.ejps.2019.105123, PMID: 31676352

