# Supplementary material for: Antimicrobial peptides: natural templates for next-generation therapeutics against antimicrobial resistance
Source: Front Cell Infect Microbiol. 2026 Jan 5;15:1720027. doi: 10.3389/fcimb.2025.1720027 (PMC12813028; doi:10.3389/fcimb.2025.1720027)
Supplement: Supplementary file 1 [file Table1.docx]

**SUPPLEMENTARY**

**Table S1**: **Time line of peptide discovery.**

| **Peptides** | **Sources** | **Year** |
| --- | --- | --- |
| Insulin | Dog pancreas | 1921 |
| Lysozyme | Saliva | 1922 |
| Gramicidin | Soil *Bacillus* strain | 1939 |
| Nisin | *Lactococcus lactis* | 1947 |
| Gramicidin A | *Brevibacillus brevis* | 1964 |
| Polymyxin (Colistin) | *Paenibacillus polymyxa* | 1947 |
| Bacillomycin | *Bacillus subtilis* | 1948 |
| Melittin | Bee venom | 1968 |
| Defensins | Rabbit lung macrophage | 1980 |
| Cecropins | *Hyalophora cecropia* | 1981 |
| Magainin | *Xenopus laevis* | 1987 |
| Thionins | Higher plants | 1985 |
| Apidaecins | *Apis mellifera* | 1989 |
| Cathelicidins | Bovine neutrophils | 1990 |
| Indolicidin | Neutrophil blood cells of cows | 1992 |
| Daptomycin | *Streptomyces roseosporus* | 1987 |
| Peptide IDR-1018 | Synthesis | 2012 |
| Teixobactin | *Eleftheria terrae* | 2015 |
| AMP-17 | *Musca domestica* | 2019 |

**Table S2**. Key structure–activity relationship (SAR) factors of antibacterial peptides are summarized, showing how charge, hydrophobicity, structure, and modifications influence potency, selectivity, and activity against GPB and GNB. The table highlights general design principles and trade-offs for optimizing peptide-based antimicrobials (Ciulla *et al*., 2023).

| **Structural Feature / Modification** | **Effect on Antibacterial Activity / Potency / Spectrum / Selectivity** | **Mechanistic or Design Insight** |
| --- | --- | --- |
| Net Positive Charge / Cationicity (Arg, Lys, His repeats, clusters) | Increased binding to negatively charged bacterial membranes (especially Gram-negative), better membrane disruption. But overly high positive charge can increase cytotoxicity. | Charge controls electrostatic interactions: needed for initial binding; balance needed to avoid toxicity. |
| Hydrophobicity & Amphipathicity | Regions of hydrophobic residues (Trp, Phe etc.) and hydrophobic patches are crucial for inserting into/melting membranes. Greater hydrophobic content often gives higher potency, especially against Gram-positive envelopes. But too much hydrophobicity → aggregation, poor solubility or toxicity to host cells. | The hydrophobic / hydrophilic balance (amphipathicity) is critical. Peptides need enough hydrophobic “face” to penetrate membrane but also enough polar/charged face for solubility / selectivity. |
| Secondary Structure (α-helix, β-sheet, cyclic / disulfide stabilized) | Helical peptides (linear) often show broad spectrum; β-sheet peptides (e.g., defensins, protegrins) need disulfide bonds to stabilize structure. Cyclic or constrained peptides are more stable to proteases and sometimes more potent. | Structural constraint *via* disulfide bridges or cyclization increases stability (proteolytic, thermal), rigidity helps retain active conformation. Also, helix vs β-sheet shapes influence membrane binding mode / pore formation / spectrum. |
| Terminal Modifications (N- / C- capping, amidation, acetylation, lipidation) | Modifications like C-terminal amidation often increase activity and stability; N-terminal acetylation sometimes improves stability, but may reduce activity for some peptides. Lipidation (adding fatty acids) can enhance membrane anchoring / insertion but may also increase toxicity or reduce solubility. | Tailoring termini helps avoid protease degradation, enhances binding to membranes, and controls peptide orientation. |
| D-amino acids and Stereochemical Changes | Replacing L-amino acids with D-amino acids increases resistance to proteolysis; sometimes these substitutions preserve or even improve antibacterial potency. However, changes in the central part of the peptide (core) often reduce activity. | Strategic placement of D-residues (e.g. at protease-sensitive positions, or termini) can boost stability while preserving function; but core stereochemistry is often critical for mechanism. |
| Disulfide Bridges / Intra-molecular Crosslinks | Disulfide bonds are essential in many β-sheet peptides (like defensins, protegrins, tachyplesins) to maintain structure and activity. Loss of disulfide bonding generally causes large loss in activity. | These stabilize the β-sheet conformation, maintain rigidity, and preserve the capability to interact properly with bacterial membranes. |
| Self-Assembly and Higher-Order Structure | Some peptides self-assemble (fibrils, micelles, etc.), which can enhance local peptide concentration at the bacterial membrane, protect susceptible peptide bonds from proteases, even modulate selectivity (less toxic to mammalian cells). | Self-assembly may be leveraged to improve stability, selectivity, and even regulatory profiles; but care is needed to avoid aggregation that impairs function or causes toxicity. |
| Glycosylation | Glycosylation in some proline-rich peptides increases specificity and modulates activity; removing glycosyl groups often reduces activity. However, the precise mechanism is less certain (possibly via receptor binding / cell wall interactions rather than membrane disruption). | Glycan moieties may assist in specificity, perhaps help binding or penetration in some bacteria, or protect peptides. But glycosylation tends to complicate manufacturing and increase cost. |
| Rigidification / Cyclic Peptides / Macrocycles | Cyclic or rigidified peptides have enhanced stability, sometimes broader spectrum, better pharmacokinetics; against resistant Gram-positives these often perform better. However, these may be harder to synthesize, and increased rigidity can reduce adaptability to different membrane types. | Rigidity ensures active conformation; but needs balance so the peptide can adapt somewhat to membrane environment. |


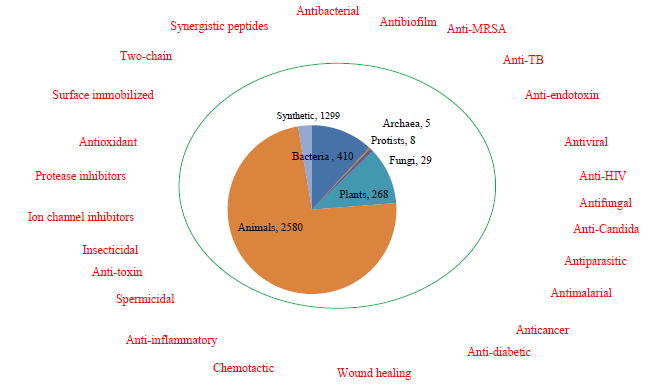


**Figure S1**: The APD3 (<https://aps.unmc.edu/>, accessed on 31 January 2025) as of January 2025, the Antimicrobial Peptide Database (APD) contains 5,099 peptides, including 3,306 natural AMPs from the six life kingdoms (410 bacteriocins/peptide antibiotics from bacteria, 5 from archaea, 8 from protists, 29 from fungi, 268 from plants, and 2,580 from animals), 1,299 synthetic, and 231 predicted AMPs.


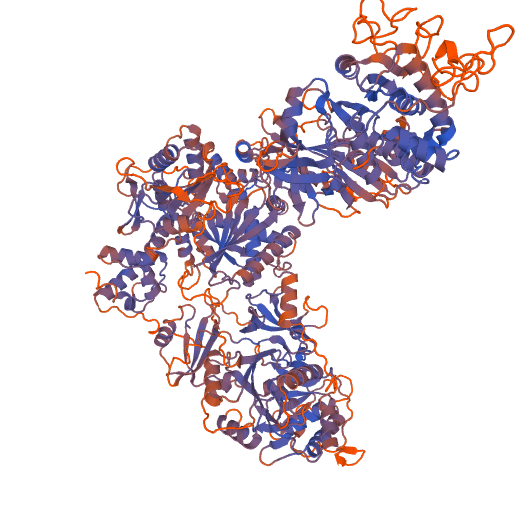


**Figure S2. The 3-D conformation model of an NRPS predicted from LC-MS data with an accession no.** [**A0A2S6P0H9**](https://www.uniprot.org/uniprot/A0A2S6P0H9). It is generated from the SWISS-MODEL template library searched with BLAST and HHBlits and deposited at ModelArchive with accession no. [ma-z4hip](https://modelarchive.org/doi/10.5452/ma-z4hip) (Ngashangva *et al*., 2021)*.*


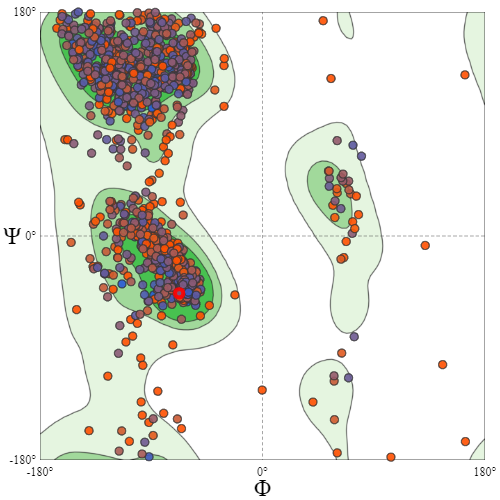


**Figure S3.** **The general Ramachandran plot and the Cβ deviation image for NRPS BGC**. The ϕ, ψ values for each residue are plotted on a background of the smoothed contours. Over 90 % lie inside the inner favoured. Ramachandran outliers is 2.36%, Rotamer outliers is 1.17%, C-Beta deviation is 19, Bad Bond is 1/13627, Bad angles is 120/18507, Cis-non proline is 3/1615, Cis-prolines is 3/82, Twisted prolines is 3/82. Molprobity score is 1.59 and Clash score is 2.26. The Gly, Pro and pre-Pro residues are on separate plots (not shown) (Ngashangva *et al*., 2021).
